# Estimating the number of genetic mutations (hits) required for carcinogenesis based on the distribution of somatic mutations

**DOI:** 10.1371/journal.pcbi.1006881

**Published:** 2019-03-07

**Authors:** Ramu Anandakrishnan, Robin T. Varghese, Nicholas A. Kinney, Harold R. Garner

**Affiliations:** 1 Gibbs Cancer Center and Research Institute, Spartanburg, SC, United States of America; 2 Department of Biomedical Sciences, Edward Via College of Osteopathic Medicine, Blacksburg, VA, United States of America; University of California Irvine, UNITED STATES

## Abstract

Individual instances of cancer are primarily a result of a combination of a small number of genetic mutations (hits). Knowing the number of such mutations is a prerequisite for identifying specific combinations of carcinogenic mutations and understanding the etiology of cancer. We present a mathematical model for estimating the number of hits based on the distribution of somatic mutations. The model is fundamentally different from previous approaches, which are based on cancer incidence by age. Our somatic mutation based model is likely to be more robust than age-based models since it does not require knowing or accounting for the highly variable mutation rate, which can vary by over three orders of magnitude. In fact, we find that the number of somatic mutations at diagnosis is weakly correlated with age at cancer diagnosis, most likely due to the extreme variability in mutation rates between individuals. Comparing the distribution of somatic mutations predicted by our model to the actual distribution from 6904 tumor samples we estimate the number of hits required for carcinogenesis for 17 cancer types. We find that different cancer types exhibit distinct somatic mutational profiles corresponding to different numbers of hits. Why might different cancer types require different numbers of hits for carcinogenesis? The answer may provide insight into the unique etiology of different cancer types.

## Author summary

Cancer is primarily a result of genetic mutations. Each individual instance of cancer is initiated by a specific combination of a small number of mutations (hits). In trying to identify these combinations of mutations, it is important to know how many hits to look for. However, there are conflicting estimates for the number of hits. We present a fundamentally different model for estimating the number of hits. We found that the number hits ranges from two-eight depending on cancer type. These findings may provide insight into the unique characteristics of different cancer types.

## Introduction

Cancer is known to result primarily from genetic mutations [1–3]. Moreover, models of carcinogenesis–the multi-stage and multi-hit models–suggest that cancer results from a small number of (two–seven) mutations [4–11]. Yet the availability of extensive genomic data [12, 13] and decades of investigation have failed to reveal, for the vast majority of cancers, the specific mutations that result in carcinogenesis. One reason for the inability to identify these carcinogenic mutations is that there is no single combination of mutations (hits) responsible for all instances of cancer, even within a cancer subtype. Instead, carcinogenesis is a result of one of many possible combinations of a small number of hits. A reliable estimate for the number of such hits will help us understand how cancers originate, and to find the specific combination of mutations responsible for individual instances of cancer [14]. However, estimates from current mathematical models are questionable because they are based on simple assumptions about mutation rate, which can vary by over three orders of magnitude as discussed below [4, 15–19].

The goal of this work is to estimate the number of genetic mutations that may be required for carcinogenesis, without making assumption about the mutation rate. We derived, from first principles, a multi-combination multi-hit mathematical model to estimate the average number of hits required for carcinogenesis. Unlike current models, our estimate is based on the distribution of somatic mutations instead of age (which requires knowing or assuming a mutation rate). In fact, we find that somatic mutations at diagnosis are weakly correlated with age at cancer diagnosis, most likely due to a large variation in mutation rates across individuals, relative to each individual’s intrinsic mutation rate. Comparing the distribution of somatic mutations calculated by the model, to the actual distribution for seventeen different cancer types, we estimate that the average number of hits varies from two to eight, depending on cancer type. Sensitivity analysis shows that, for sample size greater than 200, the distribution of somatic mutations and the corresponding estimated number of hits are robust. Some cancer types show a multimodal distribution indicating a mix of different number of hits. A key finding is that each cancer type has a distinct distribution. We discuss how the corresponding differences in the estimate for the number of hits may suggest differences in the etiology of the different cancer types.

Several factors, other than genetic mutations, may also affect carcinogenesis, such as epigenetic modifications [20], tumor environment [21], and adaptive evolution [22]. However, carcinogenesis is primarily a result of genetic mutations [23, 24]. Somatic mutations in particular are a critical driver of carcinogenesis, as exemplified by the occurrence of cancer in *Caenorhabditis elegans* and *Drosophila melanogaster*. Cancers in *C*. *elegans* are only found in the indeterminately differentiating germ-line cells and not in somatic tissues, which are terminally differentiated at birth [25]. Cancers in *D*. *melanogaster* are only found in larvae or in the gut and germline cells of adults, and not in other adult tissues with little or no cell turnover after adulthood [26, 27]. Retinoblastoma is the classic example where the disease is known to be caused by mutations to both copies of the RB1 gene, with 55–65% being non-hereditary, indicating that somatic mutations are the primary cause in these cases [28]. The causal relationship between somatic mutations and cancer is further supported by the mutagenic effects of carcinogens [29].

## Results and discussion

We present a mathematical model for predicting the distribution of somatic mutations as a function of the number of hits and combination, the multi-combination multi-hit model of carcinogenesis. The model does not make assumptions about mutation rate, which can be highly variable as suggested by the weak correlation between somatic mutations and age at cancer diagnosis. We compare the distribution calculated by the model to the actual distribution in somatic mutations to estimate the number of hits required for carcinogenesis. The estimates were robust to sample size when sample size is greater than 200. We discuss possible reasons for differences in number of hits for different cancer types. We also discuss the effect of possible deviations from model assumptions on the estimated number of hits.

### First-principles based multi-combination multi-hit model

**Fig 1:**
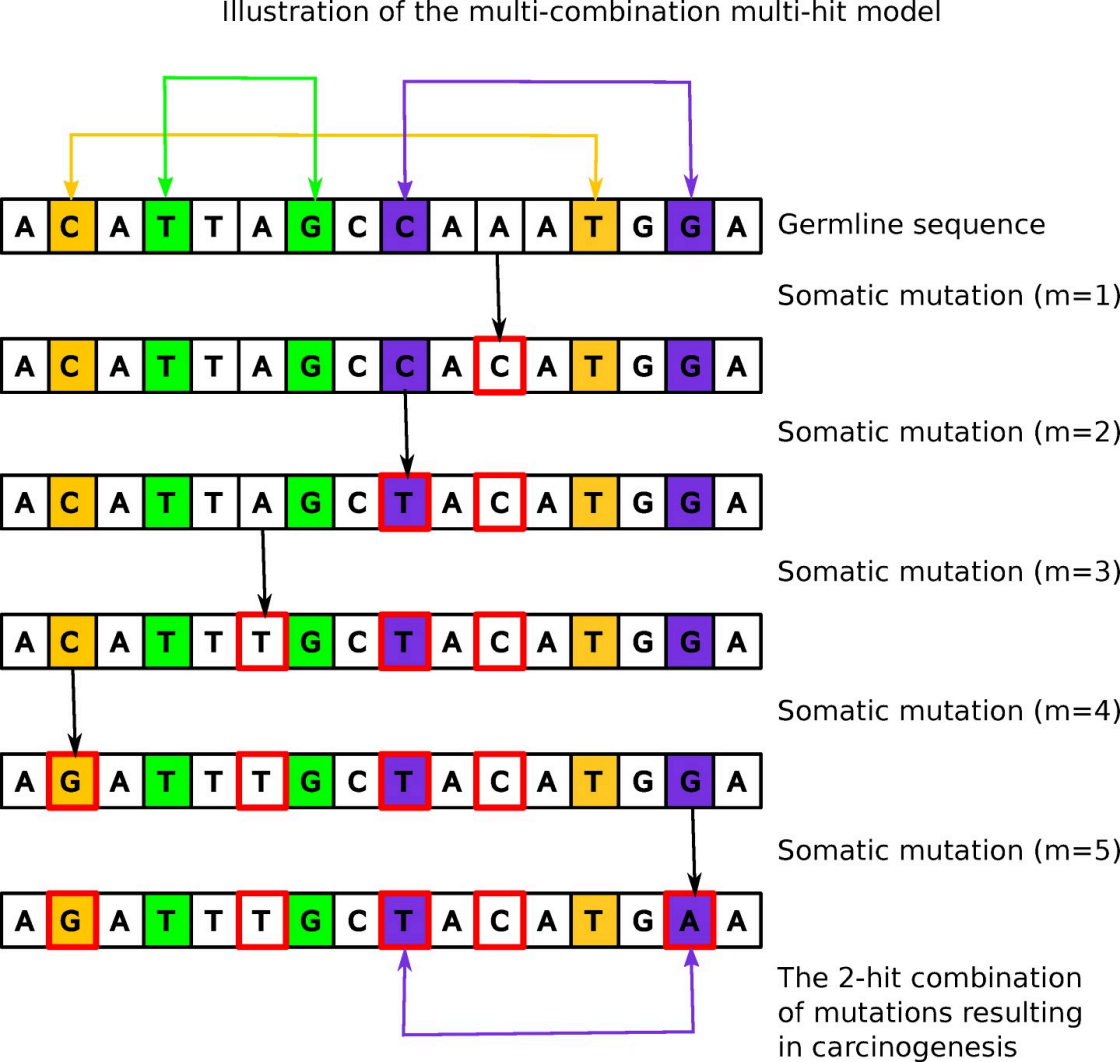
Illustration of the multi-hit model. A short genome with three (k = 3) possible 2-hit (h = 2) combinations of carcinogenic mutations. The 2-hit combinations are shown with yellow/green/purple shaded background. Somatic mutations are outlined in red. One (purple) of the three combinations occurs with five (m = 5) somatic mutations, resulting in carcinogenesis.

We derived, from first-principles (see *Materials and Methods*), a mathematical model for carcinogenesis resulting from multiple different combinations of carcinogenic mutations (hits). The probability of carcinogenesis is formulated as a function of accumulated somatic mutations, the number of hits, and the number of possible multi-hit combinations. Fig 1 illustrates the process of carcinogenesis represented by the model. The probability *P* that the *m*^th^ somatic mutation results in “hitting” one of *k* possible combinations of *h* carcinogenic mutations, can be approximated as

$$P\left(m\right)\approx P_{k}\left(m\right)-P_{k}\left(m-1\right)$$ (1)

$$P_{k}\left(m\right)\approx 1-{\left[1-P_{h}\left(m\right)\right]}^{k}$$ (2)

$$P_{h}\left(m\right)=1-\overset{h}{\underset{i=1}{\sum }}{\left(-1\right)}^{i+1}\left(\begin{array}{c} h\\ i \end{array}\right){\left[\frac{G-i}{G}\right]}^{m}\;\left(m\geq h\right)$$ (3)

where *P*_*k*_ is the cumulative probability of hitting one or more of *k* multi-hit combinations with *m* or less accumulated mutations, *P*_*h*_ is the probability of hitting one combination of *h* mutations, and *G* is the number of all possible somatic mutations that can become fixed, i.e. do not cause cell death. Here *k* is the number of possible carcinogenic multi-hit combinations, e.g. for 2-hit combinations it would include a set of genetic mutations [g_11_, g_12_], [g_21_, g_22_], … [g_k1_, g_k2_], where *g*_*ij*_ represents a genetic mutation. In this model, mutations are counted separately for each copy of a gene. The derivation for the above equations use basic principles from probability theory, as detailed in *Materials and Methods*. Although this model does not make assumptions regarding mutation rate, as current models do, we do make two other simplifying assumptions that are similar to the other assumptions made by current models [4–10]. One, mutations at any one of the loci are equally likely. This assumption does not imply that mutation rate (variants per year) is fixed. It only implies that the distribution of mutations across the genome is approximately uniform subject to random variations. Two, carcinogenesis is primarily the result of somatic mutations. We discuss below the potential effect of deviations from these assumptions, on our results.

Our model, which is based on accumulated somatic mutations, does not make any assumptions regarding mutation rate, which can be highly variable [15–19]. Whereas current models are based on incidence by age, which requires knowing or making assumptions about mutation rate, which limits the robustness of these models [4–9]. For example, the multi-stage models by Armitage and Dole, Ashley, Luebeck and Moolgavkar, Little and Wright, and Zhang and Simon, assume a fixed probability of cellular change (rate-limiting carcinogenic mutations) for each stage, and subjective explanations are provided for differences in this probability between stages [5–9]. Knudson’s 1971 study of retinoblastoma and Frank’s 2005 study of colon cancer compare incidence by age for cases with and without inherited mutations, assuming a constant mutation rate [28, 30]. Tomasetti et. al. found that mutation rates vary by a factor of three between smokers and never-smokers, and by a factor ten between those with and without a mismatch repair deficiency. They partially mitigate the effect of these variable mutation rates, by partitioning tumor samples into two subsets based on an estimated constant mutation rate for each subset [4]. Current models use age as an indirect measure of somatic mutations, which are the primary cause of cancer, i.e. *age-at-carcinogenesis = somatic-mutations-at-carcinogenesis / mutation-rate*, which requires making assumptions about the mutation rate. By directly estimating the probability of carcinogenesis as a function of somatic mutations instead of age, our model eliminates the need for making assumptions about mutation rates.

### Somatic mutations are weakly correlated with age at cancer diagnosis

**Fig 2:**
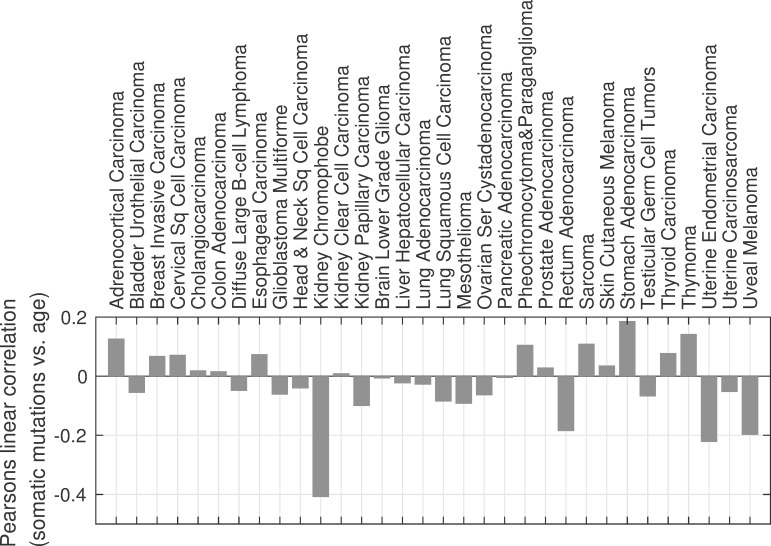
Somatic mutations at diagnosis are weakly correlated with age at cancer diagnosis. Pearson’s linear correlation between somatic mutations and age at diagnosis ranges from -0.2 to +0.2, except for kidney chromophobe for which there were only nine matched tumor and blood derived normal samples.

The number of accumulated somatic mutations in tumor samples is weakly correlated with age at cancer diagnosis, as shown in Fig 2. The Pearson’s correlation coefficient ranges from -0.2 to +0.2 for whole exome sequencing data for 31 of 32 cancer types in The Cancer Genome Atlas (TCGA) with matched tumor and blood derived normal samples. For kidney chromophobe, with correlation coefficient of -0.4, only nine samples were available. The associated p-values are greater than 0.05 for all but four of the 32 cancer types, also suggesting a lack of correlation. For breast invasive carcinoma, rectum adenocarcinoma, stomach adenocarcinoma, and uterine endometrial carcinoma, the p-values were 0.02, 0.02, 7E-4 and 4E-6 respectively, with correlation coefficients of 0.07, -0.20, 0.18, and -0.21. The calculation of p-value is described in the SI. Longitudinal studies show that for any given individual the number of accumulated somatic mutations increase with age [15]. However, this relationship can be obscured by the extensive variation in the rate of mutations between individuals. For a given individual accumulated mutations will of course increase with age. However, we find that for a population of individuals diagnosed with cancer, the correlation is weak at best. Consider for example, a smoker who is likely to get cancer at a much younger age than a nonsmoker is. It is possible that, due to the mutagenic activity of cigarette smoke, the younger smoker will have accumulated more mutations than the older nonsmoker. Some of the factors that contribute to this variation include age itself, exposure to mutagens, and mutations in DNA repair genes [4, 15–19]. Tomasetti et. al. show that the average mutation rate for smokers is 3 times higher than for never-smokers, and over ten times higher for individuals with a mismatch repair deficiency compared to those without the deficiency [4]. Tomlinson et. al. suggest that mismatch repair deficiency can increase mutation rate by up to four orders of magnitude [19]. Bavarva et. al. report that the nonsynonymous mutation rate varies by age from 9.6E-7 to 8.4E-6 per base pair per year.

### The number of carcinogenic mutations (hits) varies from two to eight depending on cancer type

**Fig 3:**
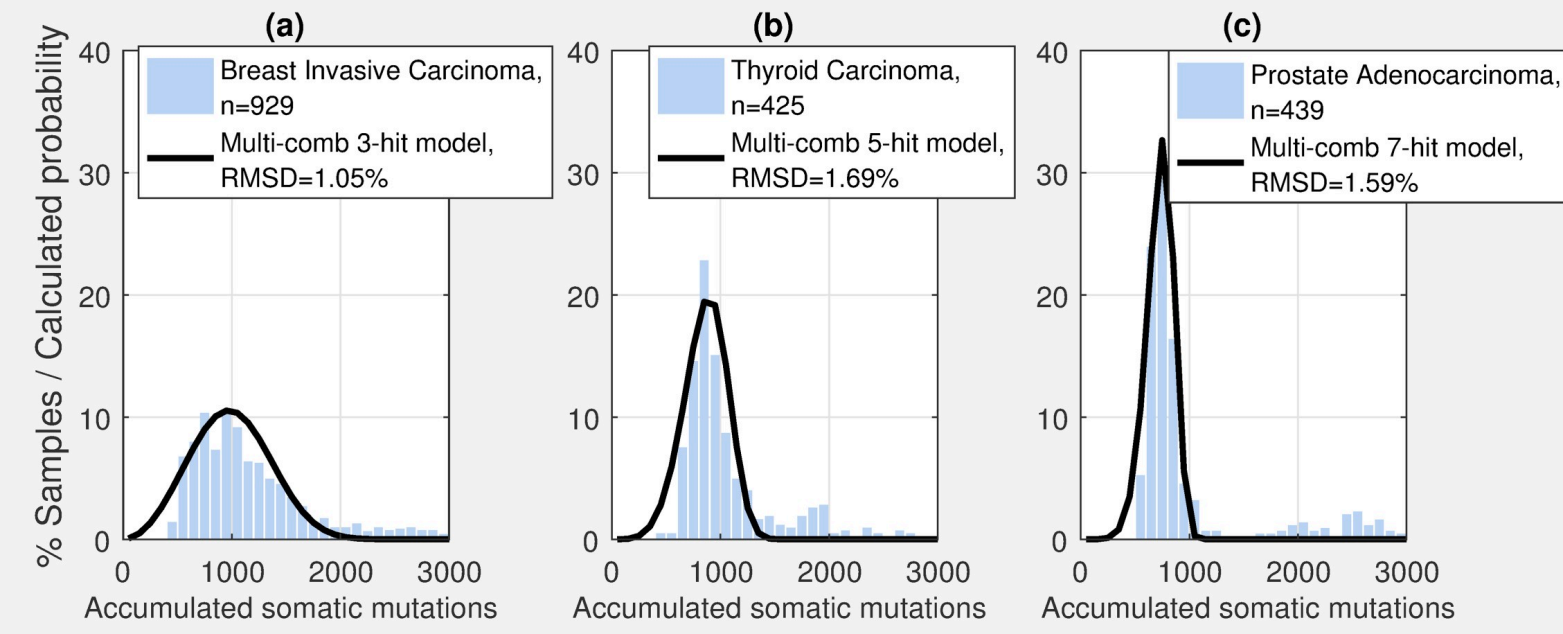
Number of hits estimated by the multi-combination multi-hit model depends on the distribution of somatic mutations. (a)-(c) Examples of three cancer types exhibiting distinct distributions and the predicted probability distribution for the optimal model, showing a corresponding difference in the number of hits. S1–S3 Figs show the distributions for the 17 cancer types with at least 200 samples.

**Fig 4:**
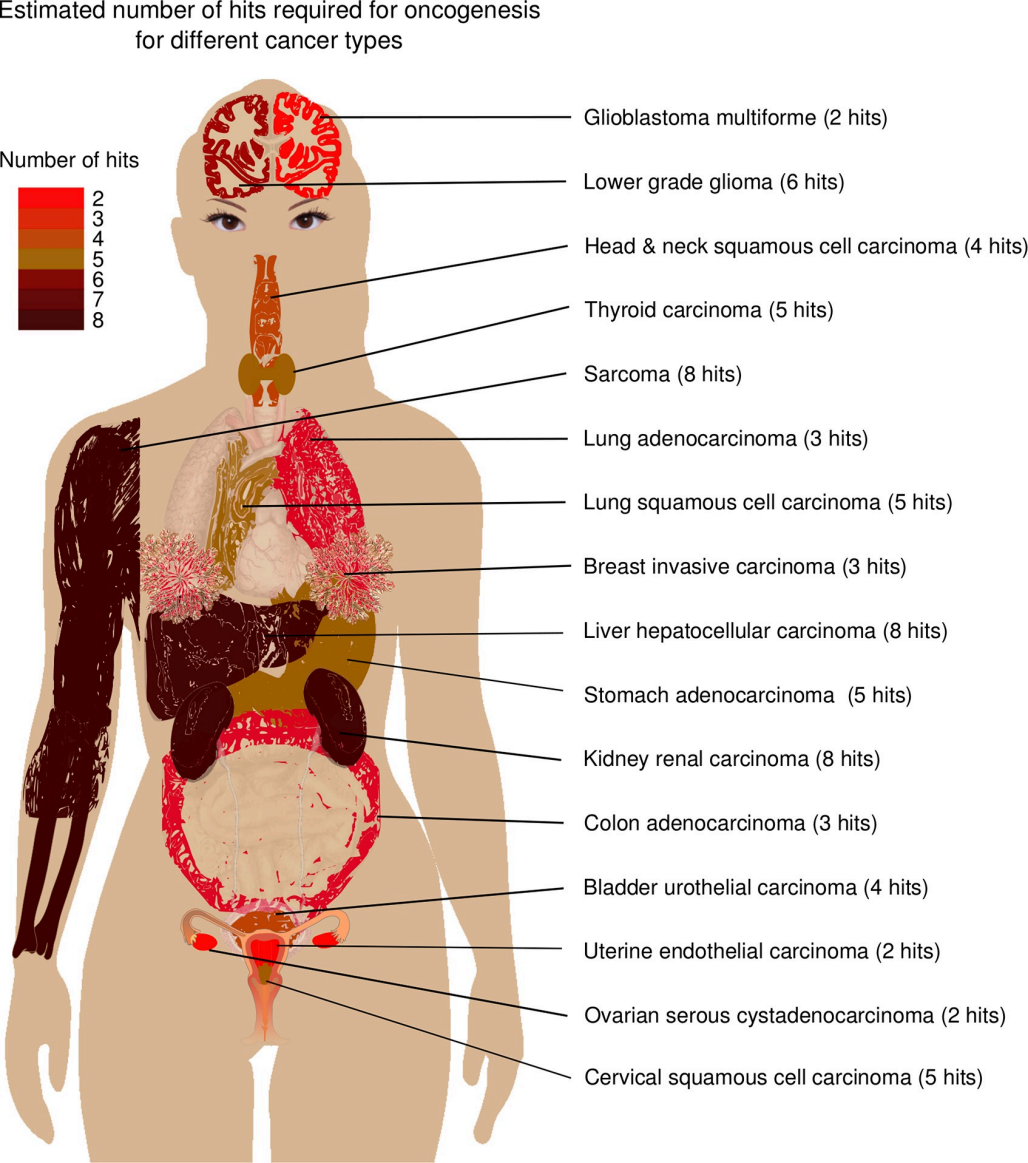
Graphical summary of estimated number of hits by cancer type. Derived from the public domain image by M Haggstrom (2014).

Comparing the actual distribution of somatic mutations for different cancer types, to the distribution of somatic mutations estimated by our model (Figs 3 and S1–S3), suggests that the average number of hits varies from two to eight depending on cancer type (Table 1, Fig 4). The number of hits were determined by minimizing root mean squared difference (RMSD) between the actual distribution of somatic mutation and the distribution calculated by the multi-combination multi-hit model (Figs 3 and S1–S3). See *Materials and Methods*. Table 1 shows that the calculated distributions closely match the actual distribution with root mean squared difference (RMSD) less than 2.2%. These results are based on whole exome sequencing data from 6904 matched tumor and blood derived normal samples for 17 of the 33 cancer types in TCGA for which there were at least 200 matched tumor and blood derived normal samples. Sensitivity analysis (next section) shows that the distribution of somatic mutations are robust for sample size greater than 200. Fig 3 shows three examples of the distribution of somatic mutations and the probability distribution calculated by the multi-combination multi-hit model. Figures for all 17 cancer types are shown in S1–S3 Figs.

**Table 1:** Number of hits estimated by the multi-combination multi-hit model range from two-eight depending on cancer type. For the 17 cancer types with at least 200 samples, the RMSD between the distribution of somatic mutations and the probability distribution for the optimal model is less than 2.2% (top section of the table). Number of hits estimated by this somatic mutations based model is in the same range as those estimated by previous models based on incidence by age (middle section). Calculation of 95% confidence interval is described in the SI. These averages may consist of a mix of different number of hits (hi and hj) as illustrated in the bottom section of the table.

Cancer Type	Glioblastoma Multiforme	Ovarian Serous Cystadenocarcinoma	Uterine Corpus Endometrial Carcinoma	Breast Invasive Carcinoma	Colon Adenocarcinoma	Lung Adenocarcinoma	Bladder Urothelial Carcinoma	Head and Neck Squamous Cell Carcinoma	Cervical Squamous Cell Carcinoma and Endocervical Adenocarcinoma	Lung Squamous Cell Carcinoma	Stomach Adenocarcinoma	Thyroid Carcinoma	Brain Lower Grade Glioma	Prostate Adenocarcinoma	Kidney Renal Papillary Cell Carcinoma	Liver Hepatocellular Carcinoma	Sarcoma
No. of samples	362	332	504	929	399	428	374	475	282	316	389	425	490	439	228	313	219
Optimal multi-comb multi-hit model																	
No. of hits	**2**	**2**	**2**	**3**	**3**	**3**	**4**	**4**	**5**	**5**	**5**	**5**	**6**	**7**	**8**	**8**	**8**
No. of comb	2x 10^9^	3x 10^9^	6x 10^9^	9x 10^14^	3x 10^14^	4x 10^14^	1x 10^20^	8x 10^19^	8x 10^24^	5x 10^24^	2x 10^25^	2x 10^25^	4x 10^30^	1x 10^36^	1x 10^40^	8x 10^39^	9x 10^39^
RMSD (%)	1.7	1.2	1.7	1.1	1.5	1.3	1.4	2.0	1.3	1.3	2.1	1.7	1.4	1.6	2.2	1.5	1.2
Estimated 95% CI	1–3	2–3	2–3	2–3	2–4	2–4	3–5	2–7	4–7	4–6	3–6	4–7	5–7	5–8	5–10	7–10	7–10
No. of hits from previous studies																	
Tomasetti et. al. 2015 *[4]*					3	3											
Zhang & Simon 2005 *[5]*				2–3													
Luebeck & Moolgavkar 2002 *[6]*					4												
Little & Wright 2003 *[7]*					5												
Ashley 1969 *[8]*	3				6		7	5	5		7	6	3				5
Armitage & Doll 1954 *[9]*		6–7	6–7	6–7	6	6–7	6–7		6–7		6–7			6–7			
Mix of two different number of hits																	
No. of hits: hi	2	2	3	3	2	3	3	3	4	4	2	3	8	6	1	3	3
No. of hits: hj	9	4	5	4	5	8	6	9	6	8	9	8	9	8	9	9	9
hi %	80	50	50	60	50	80	50	50	20	60	50	40	70	30	20	20	20
RMSD (%)	1.1	0.8	0.7	0.8	1.1	0.9	0.8	1.1	0.7	0.8	1.1	0.9	0.8	1.2	1.7	1.1	0.7

We compared our results to those of other mathematical models (Table 1). These mathematical models include both probabilistic [4, 7–9] and mechanistic models [5, 6]. The probabilistic models assume a constant mutation rate for each driver gene, which results in an exponential relationship between the probability of cancer incidence and age. The mechanistic models explicitly represent each stage of cancer progression, and the processes of cell growth, death and mutation, with a constant mutation rate for each stage. As shown in Table 1, the number of hits estimated by our model (two–eight) is approximately in the same range as previous estimates (two–seven) [4–9, 11] based on fundamentally different methodologies. More importantly the most recent, and arguably the most accurate, estimates by Tomasetti et. al. in 2015 for colon and lung cancers exactly match our estimates, lending support for the multi-combination multi-hit model, the reasonableness of the assumptions used, and the estimated number of hits.

As a further test of the robustness of our probabilistic model, we implemented and compared our results to a mechanistic model. The mechanistic model incorporates characteristics of newer mechanistic models [31–35], not included in the older mechanistic models [5, 6] considered in the comparisons shown in Table 1. The model is general enough that it can be applied to different cancer types by using an appropriate set of parameters. The details of the model are included in the SI, S5 Fig. To summarize, the model includes (a) multi-compartment spatial cellular hierarchy represented by stem cells, progenitor cells and differentiated cells, (b) initial cellular homeostasis transforming into growing tumors based on different rates of cell division, cell differentiation and cell death, and (c) selection for, and accumulation of, multiple driver mutations resulting in cancer precursor cells and then malignant cancer cells. The parameters for the model include the numbers of stem cells, progenitor cells, and differentiated cells, the rates of cell division, differentiation, and death, and the oncogenic mutation rates. We identified four cancer types (colon, lung, and stomach adenocarcinoma, and thyroid carcinoma) for which values for key model parameters were found in the literature [11, 36–39], as listed in the SI, S3 Table. When compared to the incidence of these cancer types by age from a 2013–2015 UK population study [40], the three-hit mechanistic model best fits the population study for all four cancer types (SI, S6 Fig). The estimated number of hits for the mechanistic model matches the number of hits for the probabilistic model for colon and lung adenocarcinoma and is within the 95% confidence interval for stomach adenocarcinoma (Table 1). However, the model is sensitive to the parameters used. In particular, estimates for oncogenic mutation rate vary from 10^−8^ to 10^−3^ [8, 11, 28, 41]. The number of hits estimated by the mechanistic model can exactly match our probabilistic model (Table 1) depending on the value used for this parameter (SI, S3 Table and S7 Fig). These findings provide further support for the robustness of our probabilistic model, and may give insight into, and help narrow, the range estimates for oncogenic mutation rate.

In our probabilistic model, the number of multi-hit combinations that minimize RMSD range from 2x10^9^ to 1x10^40^ depending on cancer type. This number may seem large, but is reasonable considering the much larger number of all possible combinations of mutations. The average length of a protein in human cells is estimated to be approximately 500 amino acids (1500 DNA base pairs). The Catalog of Somatic Mutations in Cancer (COSMIC) has identified over 700 confirmed cancer genes, with many more genes that have been experimentally implicated in cancer [42]. If we assume that mutations in 1000 of the 1500 loci within a gene alters the function or activity of the gene, and that altering the function or activity of any combination of *h* (number of hits) out of just 50 different genes is carcinogenic. Then, for the diploid human genome, there are approximately 10^10^ possible carcinogenic 2-hit combinations ((1000x2x50)^2^) and approximately 10^40^ possible carcinogenic 8-hit combinations.

### Results are robust for sample size greater than 200

To determine the sensitivity of our results to sample size, we estimated the number of hits using a randomly selected 80% subset of the samples. The number of matched tumor and blood derived normal samples in TCGA vary from nine for kidney chromophobe to 929 for breast invasive carcinoma. We found that for sample size greater than 200, the estimated number of hits did not change in any of the 17 cancer types, the estimated number of combinations did not change in 14 of 17 cancer types, and the change in RMSD was less than 0.11% in 16 of 17 cancer types. Results for all 32 cancer types with all samples and 80% of the samples are shown in S1 Table.

### Average number of hits may comprise a mix of different number of hits

Some of the cancer types exhibit multi-modal distributions, suggesting a mix of different number of hits, such as thyroid carcinoma (THCA) and prostate adenocarcinoma (PRAD) as seen in Fig 3(B) and 3(C). In addition, in some cases there is little difference in the RMSD between adjacent numbers of hits, also indicating a mix of different number of hits. For example, for head and neck squamous cell carcinoma (HNSC) the minimum RMSD for the 4-hit model is 2.03 compared to 2.06 for the 5-hit model. To assess the effect of a mix of different number of hits, we identified the optimal pairwise combination of hits from two to nine for each cancer type. The combined probability for a mix of *a*% hi-hits and (100-*a*)% hj-hits was calculated as *P*_*hihj* =_ (*a*.*P*_*hi*_ + (100 − *a*).*P*_*hj*_)/100. The RMSD between the actual and calculated distributions were lower in all cases with the maximum RMSD being reduced from 2.1 to 1.7 (Table 1). For THCA, for example, a mix of 40% 3-hit combinations and 60% 8-hit combinations fit the distribution with RMSD of 0.85 compared to the 5-hit combination with RMSD 1.69. For PRAD, a mix of 30% 6-hit combinations and 70% 8-hit combinations fit the distribution with RMSD of 1.2 compared to 1.6 for the 7-hit combination alone. For HNSC, a mix of 50% 3-hit and 9-hit combinations reduced the RMSD from 2.0 to 1.1. We speculate that the mix of different number of hits may represent a mix of different cancer subtypes. Determining the number of hits associated with different subtypes will require a statistically significant number of samples for each subtype, more than what is currently available in TCGA.

### The number of hits may depend on tissue specific cellular growth characteristics

Different cancer types exhibit distinct distributions for somatic mutations, as seen in Figs 3 and S1–S3. In addition, these distributions are robust for sample sizes greater than 200 (S1 Table). Corresponding to these different distributions, the number of hits estimated by our model are also different (Table 1). We speculate that there may be a relationship between the number of hits and the cellular growth characteristics of the tissue of origin for the different cancers. Previous studies have also identified tissue and cell specific differences in carcinogenesis [21, 36, 43, 44]. Tomasetti & Vogelstein linked the tissue specific variation to lifetime stem cell divisions [36, 43]. Nunney suggests that tissue specific variations may be a result of adaptive evolution, and that these adaptations may be a function of the number of tissue specific cell divisions [44]. Schneider et. al. have attributed tissue-specific difference in cancer to differences in cellular environment and signaling networks [21].

**Fig 5:**
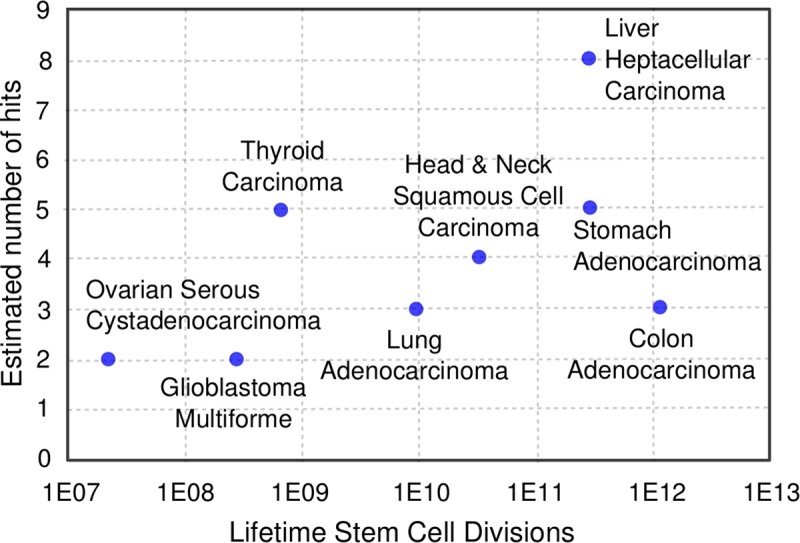
Estimated number of hits are moderately correlated to lifetime stem cell division. Pearson’s linear coefficient = 0.522, suggesting that number of hits may depend on cellular growth characteristics of individual tissues. However, the 95% confidence interval = -0.29–0.90, indicating that the relationship may be coincidental. Estimates for lifetime stem cell divisions were from S1 Table of Ref. (29).

Tomasetti and Vogelstein [43], for example, found a strong correlation (Pearson’s linear correlation = 0.804) between the incidence of different cancer types and the lifetime stem cell divisions in the corresponding tissue, highlighting the importance of tissue specific cellular growth characteristics on cancer incidence. We also found a moderate correlation (Pearson’s linear correlation = 0.52) between the estimated number of hits and lifetime stem cell division for eight cancer types that match the Tomasetti and Vogelstein classification (Fig 5). However, this relationship may be coincidental (95% confidence interval = -0.29 to 0.90) due to the small number of data point.

**Fig 6:**
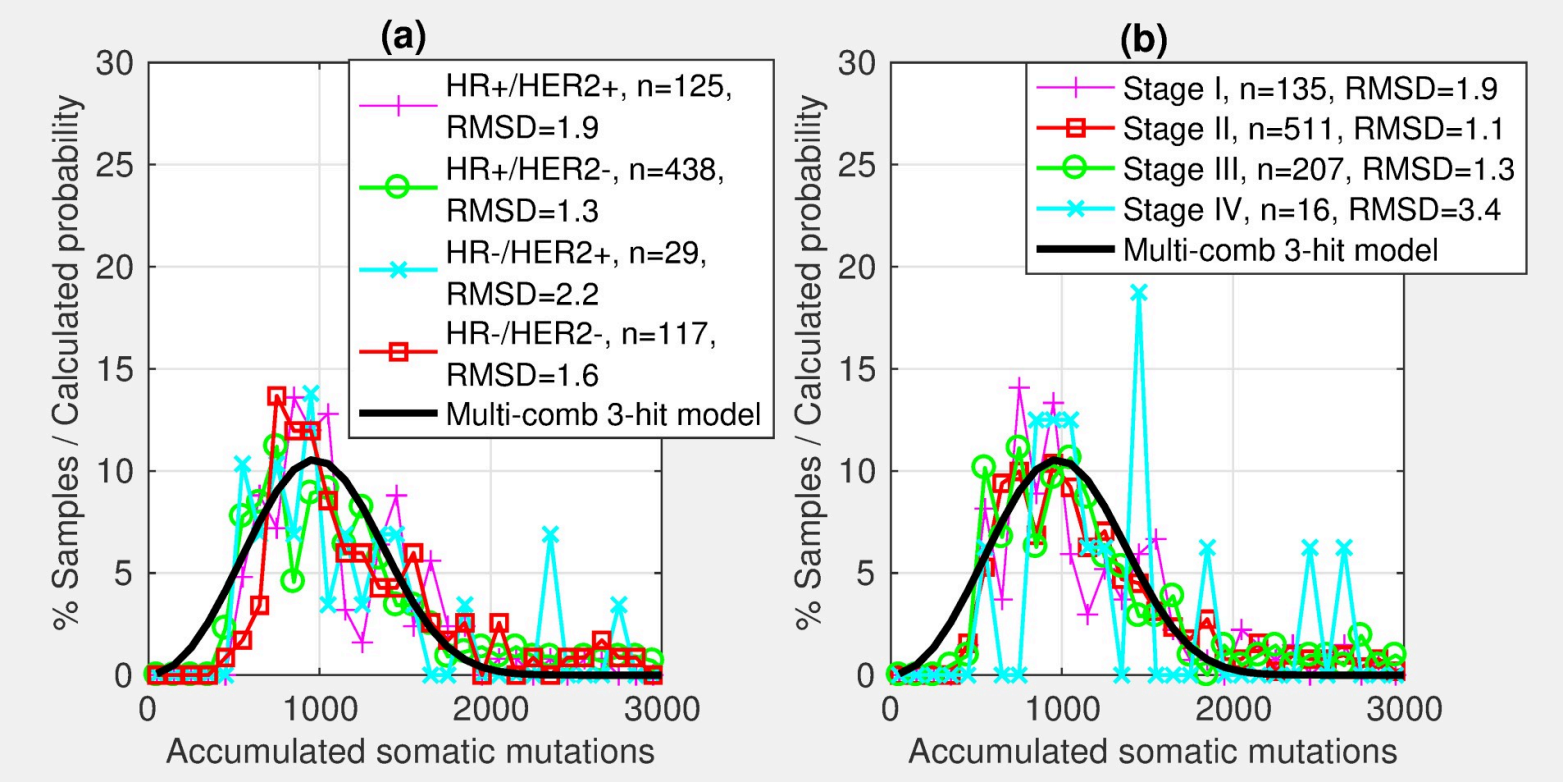
Distribution of somatic mutations for breast invasive carcinoma (BRCA) is similar by subtype and stage. The estimated number of hits is identical for subsets of BRCA samples by (a) subtype and (b) stage.

An analysis of breast invasive carcinoma (BRCA) subtypes shows that the distribution of somatic mutations are similar across subtypes (Fig 6(A)), resulting in identical optimal multi-combination multi-hit models. This again suggests that the number of hits may depend on the cellular growth characteristics of the tissue. The 709 BRCA samples, for which subtype information was available in TCGA, contain 62% hormone receptor positive (HR+) / tyrosine kinase-type cell surface receptor negative (HER2-), 17% HR-/HER2- (triple negative), 17% HR+/HER2+, and 4% HR-/HER2+ subtypes (roughly comparable to a recent study showing 73%, 12%, 10%, and 5% respectively) [45]. We subdivided the samples into four subsets corresponding to these subtypes. The estimated number of hits, and the number of multi-hit combinations was identical for all four subtypes. One possible explanation is that the number of hits may be determined by the growth characteristics of the tissue. Although the different subtypes may result from different combinations of mutations, the estimated number of hits are the same.

Another example of a possible link between the number of hits and tissue specific cellular growth characteristics are the tissues whose growth is regulated by female sex hormones: breast, ovarian and uterine epithelia [46]. These three cancers require on average only two-three hits. We speculate that since these tissues are actively growing for a relatively short time (from puberty to menopause), the associated cells may not require a more robust cell proliferation control mechanism, i.e. larger number of hits. Which may explain why continued exposure to female sex hormones past menopause increases the risk of breast, uterine and ovarian cancers [47–49].

For BRCA, the distribution of somatic mutations is also similar across the four cancers stages, corresponding to the same number of hits (Fig 6(B)), suggesting that there is no significant increase in somatic mutations with cancer stage. One possible explanation is that tumor growth originates from a relatively small number of cancer stem cells with limited subsequent differentiation [50]. A second possible explanation is that the variance in somatic mutations across individuals may obscure the relatively smaller increase in somatic mutations with cancer stage. The average somatic mutations per sample for all cancer types in TCGA for which cancer stage information was available, was 1551, 1433, 1299 and 1429 for stages I, II, III and IV respectively, also suggesting no significant increase in somatic mutations with cancer stage.

### Potential effect of deviations from model assumptions

As noted above, unlike current models, we do not make assumptions about mutation rate. However. to derive a closed-form mathematical model (Eqs (1)–(3)) for this complex biological process, we make two simplifying assumptions. These assumptions are similar to the assumptions made by current models [4–10]. (1) Somatic mutations are equally likely at any location in the genome, and (2) carcinogenesis is driven primarily by somatic mutations. While the first assumption is unlikely to affect our results, the second assumption will likely result in the number of hits being understated, as discussed below.

Contrary to assumption (1), susceptibility to mutations varies across the genome. For example, CpG sites and tandem repeat regions are known to be much more mutable than the overall average rate [51–53]. However, these regions are distributed across the genome and across genes. Therefore, although tumors may be more likely to include hits to these regions, the estimated number of hits themselves are unlikely to be affected, e.g. more of the h-hit combinations found in the tumor samples may include mutations to tandem repeat regions, but the number of hits and number of possible combinations remain the same. In addition, our results are robust to large changes in the value of *G*, the number of possible somatic mutations. S1 and S2 Tables show that, when the value of *G* is changed by a factor of eight, the estimated number of hits for all cancers with at least 100 samples remain the same. As an extreme example, if we assume that all somatic mutations only occur in 12.5% (1/8^th^) of the genome, with no somatic mutations in the rest of the genome. Then, the value of G would change by a factor of eight, and as we have shown (S1 and S2 Tables), even such an extreme change does not affect the estimated number hits. It is also unclear if the variance in mutability can be incorporated into a closed form solution, such as Eqs (1)–(3). If it could be done, any potential benefit is outweighed by the increased dimensionality and higher uncertainty due to the additional parameters needed.

Contrary to assumption (2), in addition to somatic mutations, other changes, such as germline mutations and epigenetic changes are also known to be responsible for tumor initiation. Germline mutations, in particular, are known to indicate a significant predisposition to cancer. For example, potentially pathogenic germline BRCA mutations were found in approximately 5% of ovarian cancers and in 12% of young-onset breast cancer, representing 1% of all breast cancer cases [54, 55]. If we conservatively assume that 10% of hits are present in the germline, then our model underestimates the number of hits by 10%. For example, when we estimated that BRCA could consist of a mix of 80% 3-hit and 30% 4-hit combinations (Table 1), some of the 3-hit combinations could be 4-hit combinations with one germline hit and some of the 4-hit combinations could be 5-hit combinations with one germline hit, i.e. even if you are born with one strike against you, an additional 3–4 hits are required for carcinogenesis.

The mathematical model itself does not exclude mutations to non-exome regions, such as mutations in RNA, regulatory, and untranslated regions [56]. However, due to the limited availability of whole genome sequencing data for matched tumor and blood derived normal samples, we used data from whole exome sequencing. The value of *G* in Eq(3) was set accordingly to correspond to the size of the exome. In general, we find that the number of hits is determined primarily by the variance in distribution around the mode, as seen in Fig 3. Incorporating mutations to non-exome regions will increase the mode, but it is not clear if it will significantly alter the variance about the mode. For example, when we exclude silent mutations, the variance in the distribution of protein-altering mutations is similar enough that the estimated number of hits is the same for all but one of the 17 cancers.

### Conclusions

Carcinogenesis can result from many different combinations of a small number of hits. However, identifying these specific combinations from an exponentially large number of possible combinations requires knowing with some degree of certainty the number of hits. In this study, we developed a mathematical model, from first principles, for predicting the distribution of somatic mutations at carcinogenesis. The model allows us to estimate the number of hits without knowing or making assumptions about the highly variable and difficult to estimate mutation rate, which is a critical limitation of current models. The number of hits required for carcinogenesis, estimated by our model, varies from two to eight depending on cancer type. The distinct distribution of somatic mutations for different cancer types, suggests tissue and cell specific carcinogenic mechanisms. The number of hits corresponding to these distinct distributions may provide insight into the etiology of different cancers types.

## Materials and methods

Described below are the derivation for the probability function for the multi-combination multi-hit model, and data and procedure used for estimating the number of hits.

### Probability of carcinogenesis as a function of somatic mutations

We derive here an expression for the probability of carcinogenesis for the multi-combination multi-hit model. The probability is expressed in terms of *G* the number of all possible somatic mutations that can become fixed in the human genome, *h* the number of specific mutations (hits) required for carcinogenesis, *k* the number of different combinations of *h*, and *m* the total number of somatic mutations. For the purpose of this model, we assume that: (1) mutations in any location within the genome is equally likely, and (2) carcinogenesis is primarily a result of somatic mutations. In the Results and Discussion section, we examine the potential effect of deviations from these assumptions. The probability of carcinogenesis is formulated as a function of *m*, *h*, *k* and *G*.

1. Number of all possible permutations of *m* mutations in *G*: *p*(*G*,*m*) = *G*^*m*^, with G as defined above.
2. Number of permutations of *m* mutations in *G* without (does not hit) *i* of *h* carcinogenic mutations: $p_{i}(m)=\left(\begin{array}{c} h\\ i \end{array}\right)p(G-i,m)$, where $\left(\begin{array}{c} h\\ i \end{array}\right)$ is the number of combinations of *i* in *h*, and *p*(*G*−*i*,*m*) = (*G*−*i*)^*m*^. The *G-i* term excludes permutations that hit a combination of *i* carcinogenic mutations.
3. Number of permutation with *h* hits: $p_{h}(m)=p(G,m)-\sum_{i=1}^{h}{\left(-1\right)}^{i+1}p_{i}(m)$, based on the inclusion-exclusion principle. This represents the difference between all possible permutations (step 1) and permutations that do not hit (step 2). The (-1)^i+1^ term excludes overlapping permutations, e.g. permutations that do not hit loci x and y for *i* = 2, also do not hit loci x for *i* = 1.
4. Probability of *h* hits: $P_{h}(m)=\frac{p_{h}(m)}{p(m)}=1-\sum_{i=1}^{h}{\left(-1\right)}^{i+1}(\begin{array}{c} h\\ i \end{array}){\left[\frac{G-i}{G}\right]}^{m}\;(m\leq h)$, representing the ratio of permutations that hit and all possible permutations.
5. Probability of *h* hits in one of *k* possible combinations: *P*_*k*_(*m*)≈1−[1−*P*_*h*_(*m*)]^*k*^. This relationship is an approximation because the individual combinations are not independent. However, it is a reasonable approximation since *G*≫*m*. In addition, the cumulative probability approaches 1 as *m* increases, providing support for the reasonableness of the approximation. The term in brackets represents the probability of not hitting a given combination of *h*-hits. The exponential term represents the probability of not hitting any of the *k* possible combination.
6. Probability of the last (*h*^th^) hit occurring on the last (*m*^th^) somatic mutation, *P*(*m*)≈*P*_*k*_(*m*)−*P*_*k*_(*m*−1), i.e. the difference between the probability of hitting after *m* and *m*-1 somatic mutations.

In the above derivation, there are several probability terms with distinct meanings. The lower case *p* refer to a number of permutations, while the upper case *P* refer to a probability. *p(G*, *m)* and *p(G-i*, *m)* are the standard function for the number of permutations of *m* in *G* and *G-i* respectively. *p*_*i*_*(m)* is the number of permutations where none of the *m* mutations hit *i* of the *h* carcinogenic mutations in a given h-hit combination. *p*_*h*_*(m)* is the number of permutations where all *h* of the mutations in a given h-hit combination occur with *m* mutations. *P*_*h*_*(m)* is the probability of all *h* mutations in a given h-hit combination occurring with *m* mutations. *P*_*k*_*(m)* is the probability of all *h* mutations in any one of *k* h-hit combinations occurring with *m* mutations.

### The Cancer Genome Atlas (TCGA) data used to estimate the number of hits

The TCGA data repository includes somatic mutations calculated by comparing whole exome sequencing data from matched blood derived normal and tumor tissue samples (MAF files). For this study we used the somatic mutations calculated using the MuTect2 variant caller [57]. We had access to data for 8292 samples for 32 cancer type. Of these, 6904 samples were for the 17 cancer types with over 200 samples.

### Identifying the optimal multi-combination multi-hit model

To estimate the number of hits, we compared the actual distribution of somatic mutations in TCGA to the distribution calculated by the model for different numbers of hits and combinations. The estimated number of hits is the value that minimizes the root mean squared difference (RMSD) between the actual and calculated distribution. The process consisted of the following steps:

1. For each cancer type, using MAF data from TCGA, we calculated the number of somatic mutations per sample.
2. The samples were grouped by the number of somatic mutations per sample into bins of size 100 somatic mutations, i.e. 0–100, 101–200, etc., to calculate the distribution.
3. The probability of carcinogenesis for somatic mutations ranging from 0 to 5000 was calculated using Eq(1). The value of *G* was set to 3.25x10^9^x2x1.1x1.5/100 representing ~3.25 billion base-pair diploid genome consisting of ~1.5% exome loci, with on average 1.1 possible mutations that can become fixed at each location. The factor if 1.1 is based on (a) on average one of all possible mutations at each location is synonymous and (b) an additional approximately 10% of mutations in TCGA samples were non-synonymous mutations. The number of hits was varied from *h* = 1 to 9 and the values for the number of combinations *k* was varied in the neighborhood of the local minima for each value of *h*. The ranges of values for *k* were 1E3-1E6, 1E8-1E12, 1E12-1E17, 1E16-1E22, 1E20-1E27, 1E27-1E33, 1E30-1E38, 1E34-1E43, and 1E37-1E48 for 1- to 9-hits, respectively. The calculated probability was also accumulated in bins of 100 somatic mutations for comparison to the actual distribution.
4. To identify the optimal multi-combination multi-hit model we minimized RMSD between the actual and calculated distributions.

## Supporting information

S1 Text Supporting Information Text. Description of confidence interval and p-value calculation, and details of the mechanistic model.
(PDF)

S1 Fig Number of hits estimated by the multi-combination multi-hit model depends on the distinct distribution of somatic mutations, Fig 1 of 3. (a)-(f) Six of seventeen cancer types with at least 200 matched tumor and blood derived normal samples, with two-three hits.
(EPS)

S2 Fig Number of hits estimated by the multi-combination multi-hit model depends on the distinct distribution of somatic mutations, Fig 2 of 3. (a)-(f) Six of seventeen cancer types with at least 200 matched tumor and blood derived normal samples, with four-five hits.
(EPS)

S3 Fig Number of hits estimated by the multi-combination multi-hit model depends on the distinct distribution of somatic mutations, Fig 3 of 3. (a)-(e) Five of seventeen cancer types with at least 200 matched tumor and blood derived normal samples, with six-eight hits.
(EPS)

S4 Fig Calculation of 95% confidence interval (CI) for the number of hits. The range of values for the 95% CI are calculated as describe in the SI. The TCGA codes for the cancer types are shown in S2 Table.
(EPS)

S5 Fig Mechanistic model of tumor growth. (EPS)

S6 Fig Cancer incidence probability estimated by mechanistic model and a recent UK population study. (a)- (d) Results for four cancer types for which key model parameters were found in the literature.
(TIF)

S7 Fig Cancer incidence probability estimated by mechanistic model with alternate values for oncogenic mutation rate. (a)- (d) Results for four cancer types for which key model parameters were found in the literature.
(TIF)

S1 Table Results are robust for sample size greater than 200. For sample size greater than 200, there is no difference in number of hits between results for all samples and randomly selected 80% of samples, and the number of combinations is different in only one case. Although there are no differences in the number of hits for 100–200 samples, the RMSD in many cases is large, due to significant discontinuity in the distribution.
(DOCX)

S2 Table Results are robust for different values of G, the number of possible mutations. The estimated number of hits are the same when G is 8 times the value used for the results shown in Tables 1 and S1, except for uterine carcinosarcoma (UCS).
(DOCX)

S3 Table Parameters for mechanistic model of tumor growth. (DOCX)

## References

[1] Ciriello G, Miller M, Aksoy B, Senbabaoglu Y, Schultz N, Sander C. Emerging landscape of oncogenic signatures across human cancers. Nature Genetics. 2013;45(10):1127–33. 10.1038/ng.2762 24071851 PMC4320046

[2] Hanahan D, Weinberg R. Hallmarks of Cancer: The Next Generation. Cell. 2011;144:646–74. 10.1016/j.cell.2011.02.013 21376230

[3] Futreal A, Coin L, Marshall M, Down T, Hubbard T, Wooster R, et al A census of human cancer genes. Nat Rev Cancer. 2004;4(3):177–83. 10.1038/nrc1299 14993899 PMC2665285

[4] Tomasetti C, Marchionni L, Nowak M, Parmigiani G, Vogelstein B. Only three driver gene mutations are required for the development of lung and colorectal cancers. Proc Natl Acad Sci U S A. 2015;112(1):118–23. 10.1073/pnas.1421839112 25535351 PMC4291633

[5] Zhang X, Simon R. Estimating the number of rate limiting genomic changes for human breast cancer. Breast Cancer Res Treat. 2005;91(2):121–4. 10.1007/s10549-004-5782-y . 15868439

[6] Luebeck G, Moolgavkar S. Multistage carcinogenesis and the incidence of colorectal cancer. Proc Natl Acad Sci U S A. 2002;99(23):15095–100. 10.1073/pnas.222118199 12415112 PMC137549

[7] Little MP, Wright EG. A stochastic carcinogenesis model incorporating genomic instability fitted to colon cancer data. Mathematical Biosciences. 2003;183(2):111–34. 10.1016/s0025-5564(03)00040-3 12711407

[8] Ashley DJ. The two "hit" and multiple "hit" theories of carcinogenesis. Br J Cancer. 1969;23(2):313–28. 5788039 10.1038/bjc.1969.41 PMC2008269

[9] Armitage P, Doll R. The Age Distribution of Cancer and a Multi-stage Theory of Carcinogenesis. Br J Cancer. 1954;8(1):1–12. 13172380 10.1038/bjc.1954.1 PMC2007940

[10] Nordling CO. A New Theory on the Cancer-inducing Mechanism. Br J Cancer. 1953;7(1):68–72. 10.1038/bjc.1953.8 13051507 PMC2007872

[11] Nunney L, Muir B. Peto's paradox and the hallmarks of cancer: constructing an evolutionary framework for understanding the incidence of cancer. Philos Trans R Soc Lond B Biol Sci. 2015;370(1673). 10.1098/rstb.2015.0161 26056359 PMC4581038

[12] Hudson TJ, Anderson W, Artez A, Barker AD, Bell C, Bernabe RR, et al International network of cancer genome projects. Nature. 2010;464(7291):993–8. Medline: 10.1038/nature08987 . 20393554 PMC2902243

[13] Weinstein J, Collisson E, Mills G, Shaw K, Ozenberger B, Ellrott K, et al The Cancer Genome Atlas Pan-Cancer analysis project. Nature Genetics. 2013;45(10):1113–20. 10.1038/ng.2764 24071849 PMC3919969

[14] Dash S, Kinney NA, Varghese RT, Garner HR, Feng W-c, Anandakrishnan R. Differentiating between cancer and normal tissue samples using multi-hit combinations of genetic mutations. Scientific Reports. 2019;9(1):1005 10.1038/s41598-018-37835-6 30700767 PMC6353925

[15] Bavarva JH, Tae H, McIver L, Karunasena E, Garner HR. The dynamic exome: acquired variants as individuals age. Aging (Albany NY). 2014;6(6):511–21. 10.18632/aging.100674 25063753 PMC4100812

[16] Paashuis-Lew YR, Heddle JA. Spontaneous mutation during fetal development and post-natal growth. Mutagenesis. 1998;13(6):613–7. Medline:. 9862193 10.1093/mutage/13.6.613

[17] Alexandrov L, Jones P, Wedge D, Sale J, Campbell P, Nik-Zainal S, et al Clock-like mutational processes in human somatic cells. Nature Genetics. 2015;47(12):1402–7. 10.1038/ng.3441 26551669 PMC4783858

[18] Wu XW, Strome ED, Meng QC, Hastings PJ, Plon SE, Kimmel M. A robust estimator of mutation rates. Mutat Res-Fund Mol M. 2009;661(1–2):101–9. 10.1016/j.mrfmmm.2008.11.015 WOS:000263609200013. 19100753

[19] Tomlinson IPM, Novelli MR, Bodmer WF. The mutation rate and cancer. Proc Natl Acad Sci U S A. 1996;93(25):14800–3. 8962135 10.1073/pnas.93.25.14800 PMC26216

[20] Stahl M, Kohrman N, Gore S, Kim T, Zeidan A, Prebet T. Epigenetics in Cancer: A Hematological Perspective. PLoS Genet. 2016;12(10):e1006193 10.1371/journal.pgen.1006193 27723796 PMC5065123

[21] Schneider G, Schmidt-Supprian M, Rad R, Saur D. Tissue-specific tumorigenesis: context matters. Nat Rev Cancer. 2017;17(4):239–53. 10.1038/nrc.2017.5 . 28256574 PMC5823237

[22] Almassalha L, Bauer G, Chandler J, Gladstein S, Szleifer I, Roy H, et al The Greater Genomic Landscape: The Heterogeneous Evolution of Cancer. Cancer Research. 2016;76(19):5605–9. 10.1158/0008-5472.CAN-16-0585 27550448 PMC5084919

[23] Vogelstein B, Papadopoulos N, Velculescu VE, Zhou S, Diaz LA Jr., Kinzler KW. Cancer genome landscapes. Science (New York, N Y). 2013;339(6127):1546–58. Medline: 10.1126/science.1235122 . 23539594 PMC3749880

[24] Weinberg RA. Coming full circle-from endless complexity to simplicity and back again. Cell. 2014;157(1):267–71. 10.1016/j.cell.2014.03.004 . 24679541

[25] Pinkston JM, Garigan D, Hansen M, Kenyon C. Mutations that increase the life span of C-elegans inhibit tumor growth. Science. 2006;313(5789):971–5. 10.1126/science.1121908 WOS:000239817000045. 16917064

[26] Eichenlaub T, Cohen SM, Herranz H. Cell Competition Drives the Formation of Metastatic Tumors in a Drosophila Model of Epithelial Tumor Formation. Curr Biol. 2016;26(4):419–27. 10.1016/j.cub.2015.12.042 . 26853367

[27] Suijkerbuijk SJ, Kolahgar G, Kucinski I, Piddini E. Cell Competition Drives the Growth of Intestinal Adenomas in Drosophila. Curr Biol. 2016;26(4):428–38. 10.1016/j.cub.2015.12.043 26853366 PMC4771920

[28] Knudson A. Mutation and Cancer: Statistical Study of Retinoblastoma. Proc Natl Acad Sci U S A. 1971;68(4):820–3. 5279523 10.1073/pnas.68.4.820 PMC389051

[29] Loeb LA, Bielas JH, Beckman RA. Cancers exhibit a mutator phenotype: clinical implications. Cancer Res. 2008;68(10):3551–7. 10.1158/0008-5472.CAN-07-5835 . 18483233

[30] Frank S. Age-specific incidence of inherited versus sporadic cancers: A test of the multistage theory of carcinogenesis. Proc Natl Acad Sci U S A. 2005;102(4):1071–5. 10.1073/pnas.0407299102 15657129 PMC545832

[31] Werner B, Dingli D, Traulsen A. A deterministic model for the occurrence and dynamics of multiple mutations in hierarchically organized tissues. J R Soc Interface. 2013;10(85):20130349 10.1098/rsif.2013.0349 23740488 PMC4043170

[32] Weekes SL, Barker B, Bober S, Cisneros K, Cline J, Thompson A, et al A multicompartment mathematical model of cancer stem cell-driven tumor growth dynamics. Bull Math Biol. 2014;76(7):1762–82. 10.1007/s11538-014-9976-0 24840956 PMC4140966

[33] Rodriguez-Brenes IA, Komarova NL, Wodarz D. Cancer-associated mutations in healthy individuals: assessing the risk of carcinogenesis. Cancer Res. 2014;74(6):1661–9. 10.1158/0008-5472.CAN-13-1452 . 24453004

[34] Fillon M. The mathematics of cancer metastases. J Natl Cancer Inst. 2013;105(2):75–6. 10.1093/jnci/djs642 . 23303865

[35] Altrock P, Liu L, Michor F. The mathematics of cancer: integrating quantitative models. Nature Reviews Cancer. 2015;15(12):730–45. 10.1038/nrc4029 26597528

[36] Tomasetti C, Li L, Vogelstein B. Stem cell divisions, somatic mutations, cancer etiology, and cancer prevention. Science. 2017;355(6331):1330–4. 10.1126/science.aaf9011 . 28336671 PMC5852673

[37] Flindt R. Amazing numbers in biology Berlin: Springer-Verlag; 2006 xiv, 295 p. p.

[38] Bertalanffy FD, Nagy KP. Mitotic activity and renewal rate of the epithelial cells of human duodenum. Acta Anat (Basel). 1961;45:362–70. . 13868378 10.1159/000141762

[39] Coclet J, Foureau F, Ketelbant P, Galand P, Dumont JE. Cell population kinetics in dog and human adult thyroid. Clin Endocrinol (Oxf). 1989;31(6):655–65. . 2627756 10.1111/j.1365-2265.1989.tb01290.x

[40] Cancer Research UK [Internet]. 2019. Available from: https://www.cancerresearchuk.org/health-professional/cancer-statistics/incidence.

[41] Hornsby C, Page K, Tomlinson I. What can we learn from the population incidence of cancer? Armitage and Doll revisited. The Lancet Oncology. 2007;8(11):1030–8. 10.1016/S1470-2045(07)70343-1 17976613

[42] Pleasance ED, Cheetham RK, Stephens PJ, McBride DJ, Humphray SJ, Greenman CD, et al A comprehensive catalogue of somatic mutations from a human cancer genome. Nature. 2010;463(7278):191–6. Medline: 10.1038/nature08658 . 20016485 PMC3145108

[43] Tomasetti C, Vogelstein B. Variation in cancer risk among tissues can be explained by the number of stem cell divisions. Science. 2015;347(6217):78–81. 10.1126/science.1260825 25554788 PMC4446723

[44] Nunney L. Commentary: The multistage model of carcinogenesis, Peto's paradox and evolution. International Journal of Epidemiology. 2016;45(3):649–53. 10.1093/ije/dyv201 26659656

[45] Howlader N, Altekruse SF, Li CI, Chen VW, Clarke CA, Ries LA, et al US incidence of breast cancer subtypes defined by joint hormone receptor and HER2 status. J Natl Cancer Inst. 2014;106(5). 10.1093/jnci/dju055 24777111 PMC4580552

[46] Johnson LR, Byrne JH. Essential medical physiology 3rd ed. Amsterdam; Boston: Elsevier Academic Press; 2003 xvi, 1008 p. p.

[47] Berrino F, Muti P, Micheli A, Bolelli G, Krogh V, Sciajno R, et al Serum sex hormone levels after menopause and subsequent breast cancer. J Natl Cancer Inst. 1996;88(5):291–6. . 8614008 10.1093/jnci/88.5.291

[48] Cramer DW, Welch WR. Determinants of ovarian cancer risk. II. Inferences regarding pathogenesis. J Natl Cancer Inst. 1983;71(4):717–21. Medline:. 6578367

[49] Smith DC, Prentice R, Thompson DJ, Herrmann WL. Association of exogenous estrogen and endometrial carcinoma. N Engl J Med. 1975;293(23):1164–7. Medline: 10.1056/NEJM197512042932302 . 1186789

[50] Kreso A, Dick JE. Evolution of the cancer stem cell model. Cell Stem Cell. 2014;14(3):275–91. 10.1016/j.stem.2014.02.006 . 24607403

[51] Pleasance E, Cheetham K, Stephens P, McBride D, Humphray S, Greenman C, et al A comprehensive catalogue of somatic mutations from a human cancer genome. Nature. 2010;463(7278):191–6. 10.1038/nature08658 20016485 PMC3145108

[52] Cooper DN, Youssoufian H. The CpG dinucleotide and human genetic disease. Hum Genet. 1988;78(2):151–5. . 3338800 10.1007/BF00278187

[53] Kinney N, Varghese RT, Anandakrishnan R, Garner HRS. ZDHHC3 as a Risk and Mortality Marker for Breast Cancer in African American Women. Cancer Inform. 2017;16:1176935117746644 10.1177/1176935117746644 29276372 PMC5734450

[54] Berchuck A, Heron KA, Carney ME, Lancaster JM, Fraser EG, Vinson VL, et al Frequency of germline and somatic BRCA1 mutations in ovarian cancer. Clin Cancer Res. 1998;4(10):2433–7. . 9796975

[55] Copson ER, Maishman TC, Tapper WJ, Cutress RI, Greville-Heygate S, Altman DG, et al Germline BRCA mutation and outcome in young-onset breast cancer (POSH): a prospective cohort study. Lancet Oncol. 2018;S1470–2045(17):30891–4. 10.1016/S1470-2045(17)30891-4 PubMed PMID: 29337092. PMC5805863 29337092

[56] Khurana E, Fu Y, Chakravarty D, Demichelis F, Rubin M, Gerstein M. Role of non-coding sequence variants in cancer. Nature Reviews Genetics. 2016;17(2):93–108. 10.1038/nrg.2015.17 26781813

[57] Cibulskis K, Lawrence MS, Carter SL, Sivachenko A, Jaffe D, Sougnez C, et al Sensitive detection of somatic point mutations in impure and heterogeneous cancer samples. Nature biotechnology. 2013;31(3):213–9. 10.1038/nbt.2514 Medline:. 23396013 PMC3833702

