# Supplementary material for: Estimating the number of genetic mutations (hits) required for carcinogenesis based on the distribution of somatic mutations
Source: PLoS Comput Biol. 2019 Mar 7;15(3):e1006881. doi: 10.1371/journal.pcbi.1006881 (PMC6424461; doi:10.1371/journal.pcbi.1006881)
Supplement: S1 Text — Description of confidence interval and p-value calculation, and details of the mechanistic model. (PDF) [file pcbi.1006881.s001.pdf]

Estimating the number of genetic mutations (hits) required for carcinogenesis based on the distribution of somatic mutations

Ramu Anandakrishnan, Robin T. Varghese, Nicholas A. Kinney, and Harold R. Garner

Supplementary Information

Included here are additional details, Figs. S1-S4 and Tables S1 and S2, in support of the main text.

Estimated number of hits

Figures S1-S3 show the h-hit model that best fits the actual distribution of somatic mutations for 17 cancer types with at least 200 samples in the cancer genome atlas (TCGA). These results are summarized in Table S1. Table S1 also shows the results from using only 80% of the available samples. The estimated number of hits is the same, indicating that the model is robust. We also show that the model is robust to model parameters. Changing the value of G, the number of possible mutations, by a factor of 8, changes the estimate for only one of the cancer types (Table S2).

Calculation of 95% confidence interval for number of hits

The confidence interval (CI) for the number of hits, shown in Table 1 of the main text, is based on Wald's CI. The range of values for h are the hits for which the root mean square difference (RMSD) is within the range $s \pm 1.96\, s/\sqrt{N}$, where s is the minimum RMSD, 1.96 is the Wald test statistic for 95% CI, and N is the number of samples. The range of hits that fall within the 95% CI are shown in Fig. S4.

Calculation p-value for correlation coefficient

The p-value for the Pearson's correlation coefficients shown in Fig. 2 of the main text are calculated as $p = T\left(\frac{r\sqrt{N-2}}{\sqrt{1-r^2}}\right)$, where p is the p-value, r is Pearson's correlation coefficient, N is the number of samples, and T is the percentage points (probability) function for the 2-tailed student t-distribution.

Mechanistic model

To further test the robustness of the our probabilistic model, we implemented and compared our results to a mechanistic model (Fig. S5). The model consists of three cell types representing the hierarchical organization of stem, progenitor and differentiated cells incorporating characteristics of newer mechanistic models [2-6]. Each of these cell types can contain up to M oncogenic mutations, represented by the 3(M+1) cell subtypes as shown in Fig. S5. N^i_j is the number of cells of type j (j=(s)tem/(p)rogenitor/(d)ifferentiated cells) with i = 0 to M mutations. The model incorporates four types of cellular transitions with rates r^i_k , where k=ss/pp/dd represents stem/progenitor/differentiated cell divisions, k=sp/pd represents stem/progenitor cell differentiation into progenitor/differentiated cells, k=d- represents cell death, and k=sm/pm/dm represents an oncogenic mutation to

stem/progenitor/differentiated cells. The following set of equations determine the population of cell subtypes shown in Fig. S5:

$$\frac{dN_s^i}{dt} = r_{ss}^i N_s^i - r_{sp}^i N_s^i - r_{sm}^i N_s^i \qquad \text{for } i = 0 \tag{1}$$

$$\frac{dN_s^i}{dt} = r_{ss}^i N_s^i + r_{sm}^{i-1} N_s^{i-1} - r_{sp}^i N_s^i - r_{sm}^i N_s^i \qquad \text{for } i > 0 \tag{2}$$

$$\frac{dN_p^i}{dt} = r_{pp}^i N_p^i + r_{sp}^i N_s^i - r_{pd}^i N_p^i - r_{pm}^i N_p^i \qquad \text{for } i = 0 \tag{3}$$

$$\frac{dN_p^i}{dt} = r_{pp}^i N_p^i + r_{sp}^i N_s^i + r_{pm}^{i-1} N_p^{i-1} - r_{pd}^i N_p^i - r_{pm}^i N_p^i \qquad \text{for } i > 0 \tag{4}$$

$$\frac{dN_d^i}{dt} = r_{dd}^i N_d^i + r_{pd}^i N_p^i - r_{d-}^i N_d^i - r_{dm}^i N_d^i \qquad \text{for } i = 0 \tag{5}$$

$$\frac{dN_d^i}{dt} = r_{dd}^i N_d^i + r_{pd}^i N_p^i + r_{dm}^{i-1} N_d^{i-1} - r_{d-}^i N_d^i - r_{dm}^i N_d^i \qquad \text{for } i > 0 \tag{6}$$

We identified four cancer types – colon, lung, and stomach adenocarcinoma, and thyroid carcinoma - for which we were able to find the following parameters in the literature. The number of stem cells (N_s^0), the number of differentiated cells (N_d^0) and the rate of stem cell division (r_{ss}^i) for all four cancer types from Tomasetti, Li and Vogelstein (2017) [7]. The differentiated cell renewal rate (r_{d-}^i) for lung and stomach from Flindt (2006) [8], for colorectal epithelial cells from Bertalaffy and Nagy (1961) [9], and for thyroid from Coclet et. al. (1989) [10]. Since the corresponding information for progenitor cells were not available, we assumed $N_p^0 = N_d^0$ and $r_{pd}^i = r_{sp}^i$. The values for r_{sp}^i, r_{pp}^i, and r_{dd}^i were set to ensure cellular homeostasis. The oncogenic mutation rate ($r_{sm}^i = r_{pm}^i = r_{dm}^i = r_{mut}$) from Nunney and Muir (2015) [11]. The parameter values used are listed in Table S3. The population of each cell subtype as a function of time was estimated by a fixed time step (dt=0.0001 years) deterministic simulation using the above equations. The estimated probability of cancer incidence by age and number of hits was then compared to a UK population study of cancer incidence [1], to estimate the number of hits required for oncogenesis. For the parameters used, we estimated the number of hits to be three for all four cancer types (Fig. S6). This estimate matches the estimate from our probabilistic model for colon and lung adenocarcinoma, and is within the 95% confidence interval estimate for stomach cancer (Table 1 of the main text). However, our model is sensitive to the value of oncogenic mutation rate, and the literature contains a wide range of value for this parameter, from 10^{-8} to 10^{-3} [11-14]. Since the set of possible cancer driver genes is diverse, with different sizes, different CpG content, and different oncogenic mutations within them, the oncogenic mutation rate is likely to vary by cancer type. Using a different set of oncogenic mutation rates, the estimated number of hits for this mechanistic model match the estimates for the probabilistic model (Table S3, Fig. S7).

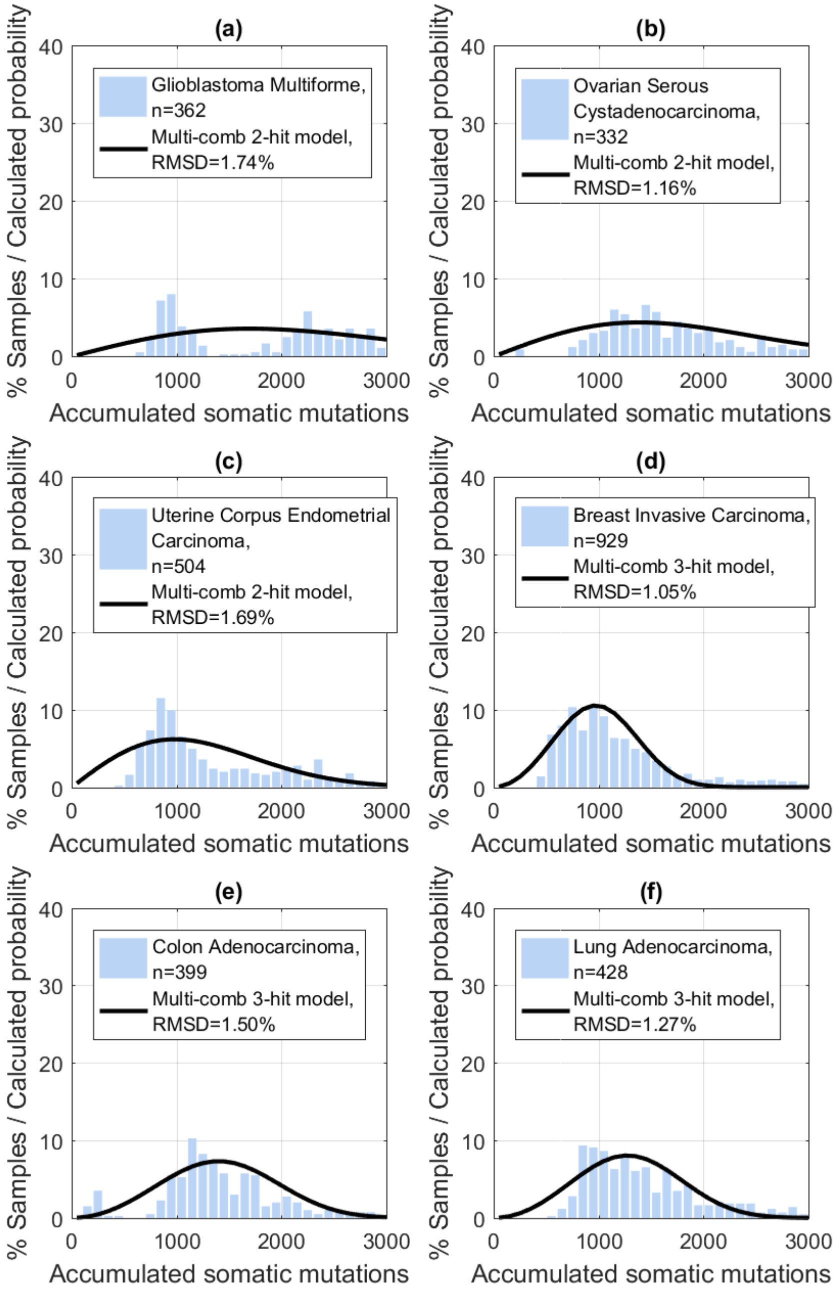

Figure S1. Number of hits estimated by the multi-combination multi-hit model depends on the distinct distribution of somatic mutations, Fig 1 of 3. *(a)-(f) Six of seventeen cancer types with at least 200 matched tumor and blood derived normal samples, with two-three hits.*

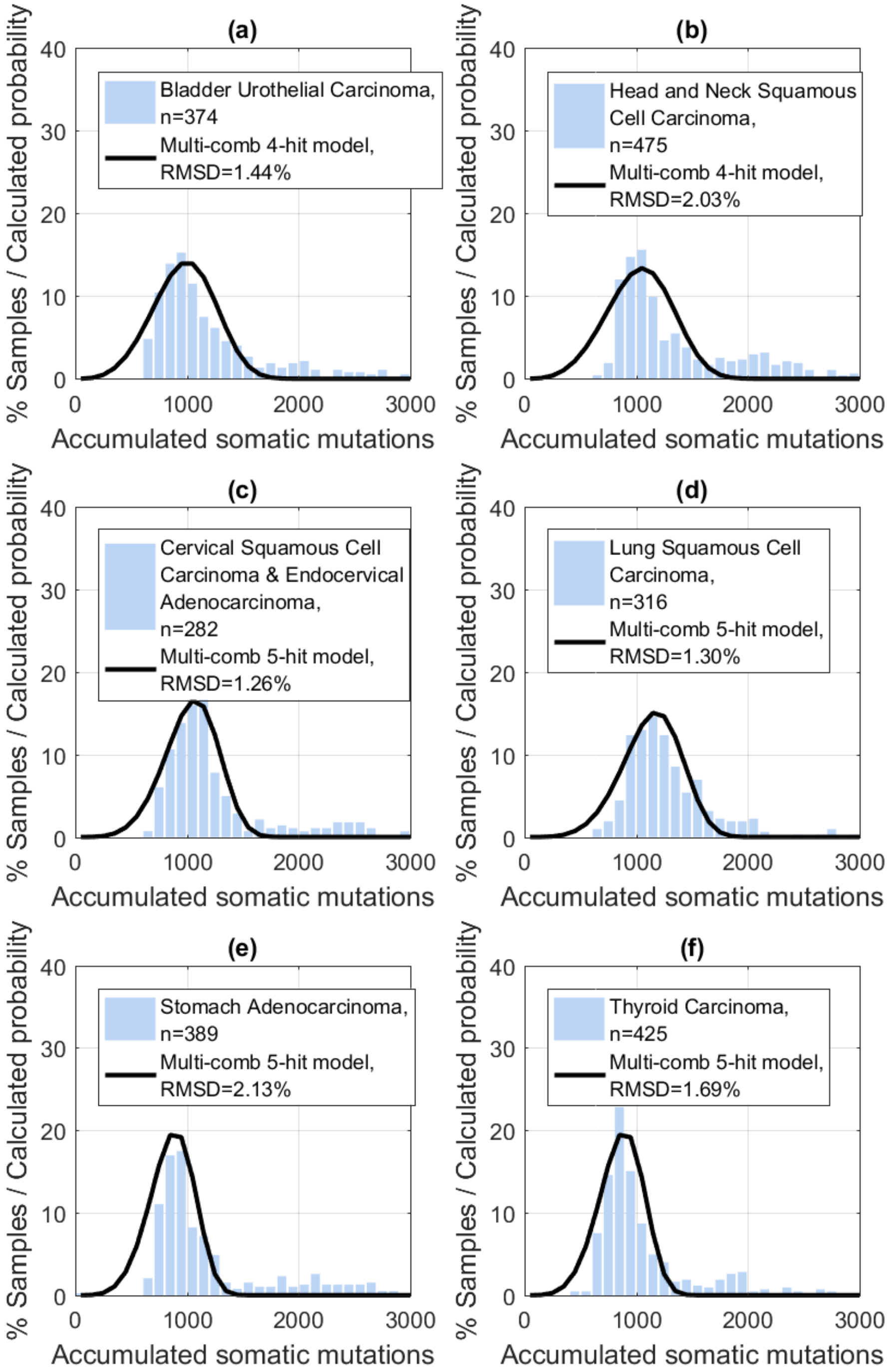

***Figure S2. Number of hits estimated by the multi-combination multi-hit model depends on the distinct distribution of somatic mutations, Fig 2 of 3**. (a)-(f) Six of seventeen cancer types with at least 200 matched tumor and blood derived normal samples, with four-five hits.*

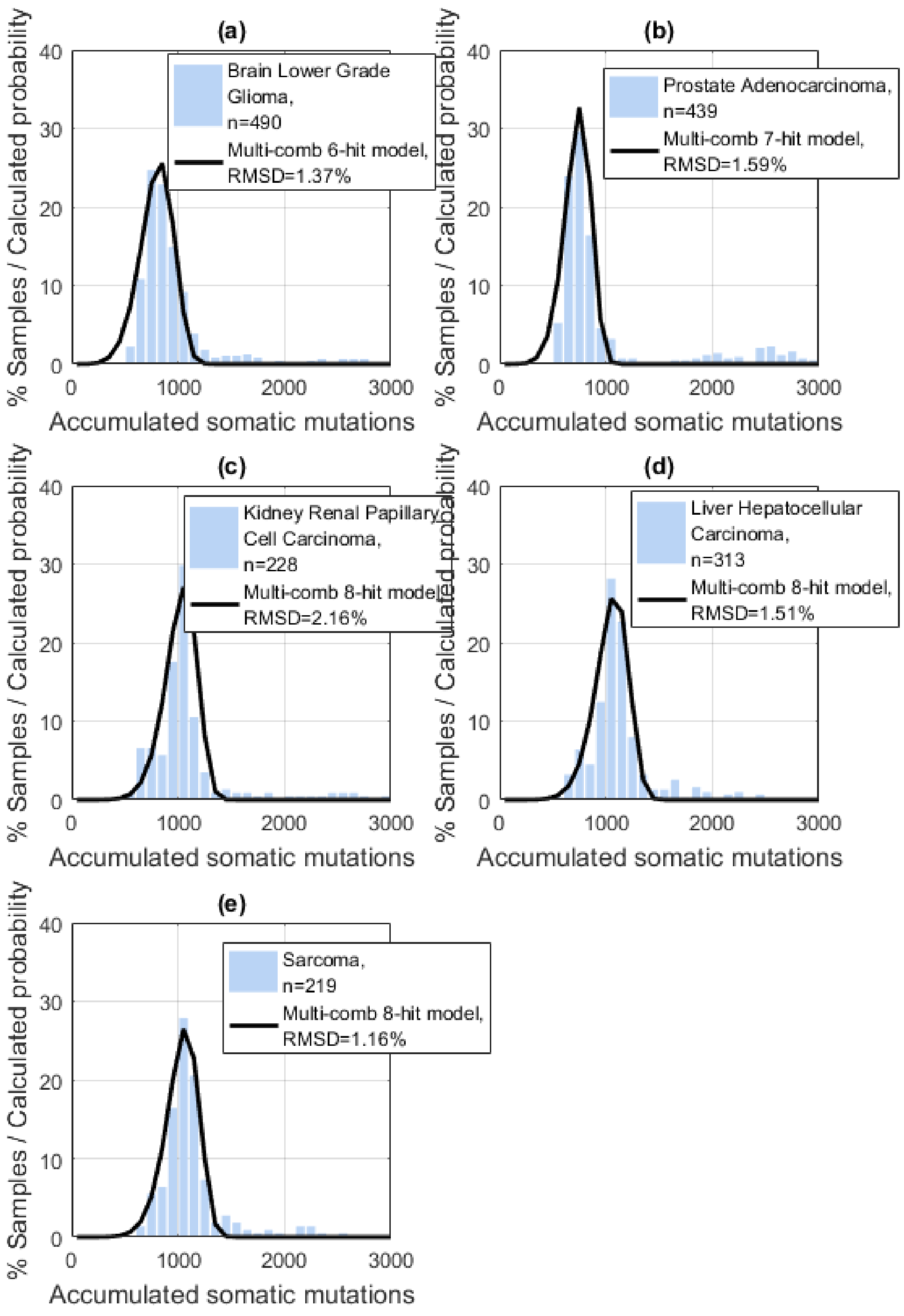

Figure S3. Number of hits estimated by the multi-combination multi-hit model depends on the distinct distribution of somatic mutations, Fig 3 of 3*. (a)-(e) Five of seventeen cancer types with at least 200 matched tumor and blood derived normal samples, with six-eight hits.*

Table S1. Results are robust for sample size greater than 200. *For sample size greater than 200, there is no difference in number of hits between results for all samples and randomly selected 80% of samples, and the number of combinations is different in only three cases. Although there are no differences in the number of hits for 100-200 samples, the RMSD in many cases is large, due to significant discontinuity in the distribution.*

TCGA code	Cancer Type	All samples				80% of samples				Difference		
		No. of samples	No. of hits	No. of combs	RMSD (%)	No. of samples	No. of hits	No. of Combs	RMSD (%)	No. of hits	No. of Combs	RMSD (%)
KICH	Kidney Chromophobe	9	9	3E+45	3.90	8	9	3E+45	4.59	0	0	-0.68
DLBC	Lymphoid Neoplasm Diffuse Large B-cell Lymphoma	44	7	4E+34	2.42	36	6	5E+29	2.56	1	4E+34	-0.14
CHOL	Cholangiocarcinoma	44	9	1E+45	3.28	32	6	6E+29	3.32	3	1E+45	-0.04
UCS	Uterine Carcinosarcoma	46	9	3E+46	1.81	33	9	3E+46	1.93	0	0	-0.12
MESO	Mesothelioma	74	9	5E+45	1.74	58	9	5E+45	1.63	0	0	0.11
UVM	Uveal Melanoma	76	9	8E+46	2.72	59	9	8E+46	2.68	0	0	0.04
ACC	Adrenocortical Carcinoma	80	9	3E+46	1.70	60	9	3E+46	1.83	0	0	-0.12
KIRC	Kidney Renal Clear Cell Carcinoma	92	3	6E+14	2.05	73	4	6E+19	2.46	-1	-6E+19	-0.40
SKCM	Skin Cutaneous Melanoma	92	3	6E+14	1.72	78	3	6E+14	1.83	0	0	-0.11
THYM	Thymoma	107	9	5E+44	1.76	87	9	5E+44	1.99	0	0	-0.23
ESCA	Esophageal Carcinoma	122	7	6E+34	1.27	92	7	6E+34	0.90	0	0	0.36
READ	Rectum Adenocarcinoma	145	5	5E+24	2.18	118	5	5E+24	2.20	0	0	-0.02
PAAD	Pancreatic Adenocarcinoma	149	9	2E+44	2.10	127	9	2E+44	2.27	0	0	-0.17
TGCT	Testicular Germ Cell Tumors	150	9	3E+45	2.54	121	9	3E+45	2.73	0	0	-0.18
PCPG	Pheochromocytoma and Paraganglioma	158	9	4E+46	2.42	126	9	4E+46	2.33	0	0	0.09
SARC	Sarcoma	219	8	9E+39	1.16	164	8	9E+39	1.50	0	0	-0.34
KIRP	Kidney Renal Papillary Cell Carcinoma	228	8	1E+40	2.16	189	8	1E+40	2.07	0	0	0.09
CESC	Cervical Squamous Cell Carcinoma and Endocervical Adenocarcinoma	282	5	8E+24	1.26	211	5	9E+24	1.35	0	-1E+24	-0.09
LUSC	Lung Squamous Cell Carcinoma	316	5	5E+24	1.30	250	5	5E+24	1.35	0	0	-0.06
LIHC	Liver Hepatocellular Carcinoma	313	8	8E+39	1.51	257	8	8E+39	1.51	0	0	0.01
OV	Ovarian Serous Cystadenocarcinoma	332	2	3E+09	1.16	279	2	3E+09	1.17	0	0	-0.01
GBM	Glioblastoma Multiforme	362	2	2E+09	1.74	285	2	2E+09	1.76	0	0	-0.03
BLCA	Bladder Urothelial Carcinoma	374	4	1E+20	1.44	299	4	1E+20	1.43	0	0	0.01
COAD	Colon Adenocarcinoma	399	3	3E+14	1.50	322	3	3E+14	1.47	0	0	0.03
STAD	Stomach Adenocarcinoma	389	5	2E+25	2.13	316	4	1E+20	2.06	1	2E+25	0.07
LUAD	Lung Adenocarcinoma	428	3	4E+14	1.27	340	3	5E+14	1.30	0	-1E+14	-0.03
PRAD	Prostate Adenocarcinoma	439	7	1E+36	1.59	363	7	1E+36	1.56	0	0	0.03
THCA	Thyroid Carcinoma	425	5	2E+25	1.69	347	5	2E+25	1.76	0	0	-0.07
HNSC	Head and Neck Squamous Cell Carcinoma	475	4	8E+19	2.03	355	4	8E+19	1.95	0	0	0.08
LGG	Brain Lower Grade Glioma	490	6	4E+30	1.37	402	6	4E+30	1.25	0	0	0.11
UCEC	Uterine Corpus Endometrial Carcinoma	504	2	6E+09	1.69	420	2	6E+09	1.74	0	0	-0.05
BRCA	Breast Invasive Carcinoma	929	3	9E+14	1.05	757	3	9E+14	1.04	0	0	0.01
Total	All cancer types	8292	3	9E+14	1.23	6664	3	9E+14	1.23	0	0	0.01

Table S2. Results are robust for different values of G, the number of possible mutations*. The estimated number of hits are the same when G is 8 times the value used for the results shown in Tables 1 and S1, except for uterine carcinosarcoma (UCS).*

TCGA code	Cancer Type	All samples			
		No. of samples	No. of hits	No. of combs	RMSD (%)
KICH	Kidney Chromophobe	9	9	2E+53	3.98
DLBC	Lymphoid Neoplasm Diffuse Large B-cell Lymphoma	44	7	4E+40	2.44
CHOL	Cholangiocarcinoma	44	9	6E+52	3.29
UCS	Uterine Carcinosarcoma	46	8	1E+48	2.27
MESO	Mesothelioma	74	9	3E+53	1.70
UVM	Uveal Melanoma	76	9	4E+54	2.76
ACC	Adrenocortical Carcinoma	80	9	1E+54	2.14
KIRC	Kidney Renal Clear Cell Carcinoma	92	3	2E+17	2.05
SKCM	Skin Cutaneous Melanoma	92	3	2E+17	1.75
THYM	Thymoma	107	9	3E+52	1.78
ESCA	Esophageal Carcinoma	122	7	7E+40	1.27
READ	Rectum Adenocarcinoma	145	5	1E+29	2.18
PAAD	Pancreatic Adenocarcinoma	149	9	9E+51	1.96
TGCT	Testicular Germ Cell Tumors	150	9	2E+53	2.63
PCPG	Pheochromocytoma and Paraganglioma	158	9	2E+54	2.41
SARC	Sarcoma	219	8	7E+46	1.16
KIRP	Kidney Renal Papillary Cell Carcinoma	228	8	9E+46	2.04
CESC	Cervical Squamous Cell Carcinoma and Endocervical Adenocarcinoma	282	5	2E+29	1.37
LUSC	Lung Squamous Cell Carcinoma	316	5	1E+29	1.29
LIHC	Liver Hepatocellular Carcinoma	313	8	6E+46	1.51
OV	Ovarian Serous Cystadenocarcinoma	332	2	1E+11	1.21
GBM	Glioblastoma Multiforme	362	2	8E+10	1.70
BLCA	Bladder Urothelial Carcinoma	374	4	3E+23	1.43
COAD	Colon Adenocarcinoma	399	3	1E+17	1.56
STAD	Stomach Adenocarcinoma	389	5	3E+29	1.96
LUAD	Lung Adenocarcinoma	428	3	2E+17	1.33
PRAD	Prostate Adenocarcinoma	439	7	1E+42	1.68
THCA	Thyroid Carcinoma	425	5	4E+29	1.70
HNSC	Head and Neck Squamous Cell Carcinoma	475	4	2E+23	2.05
LGG	Brain Lower Grade Glioma	490	6	5E+35	1.32
UCEC	Uterine Corpus Endometrial Carcinoma	504	2	3E+11	1.69
BRCA	Breast Invasive Carcinoma	929	3	3E+17	1.08
All	All cancer types	8292	3	3E+17	1.28

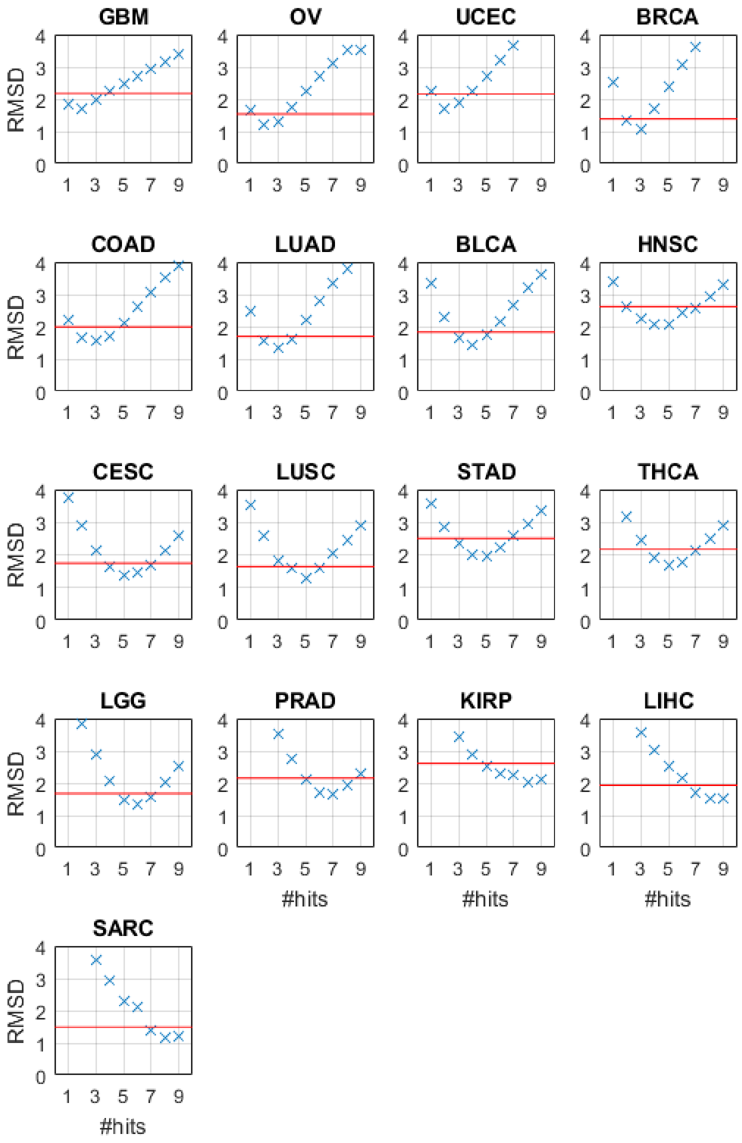

*Figure S4. **Calculation of 95% confidence interval (CI) for the number of hits.** The red line represents the RMSD value for 95% CI. The range of values for the 95% CI are calculated as describe in the SI. The TCGA codes for the cancer types are shown in Table S2.*

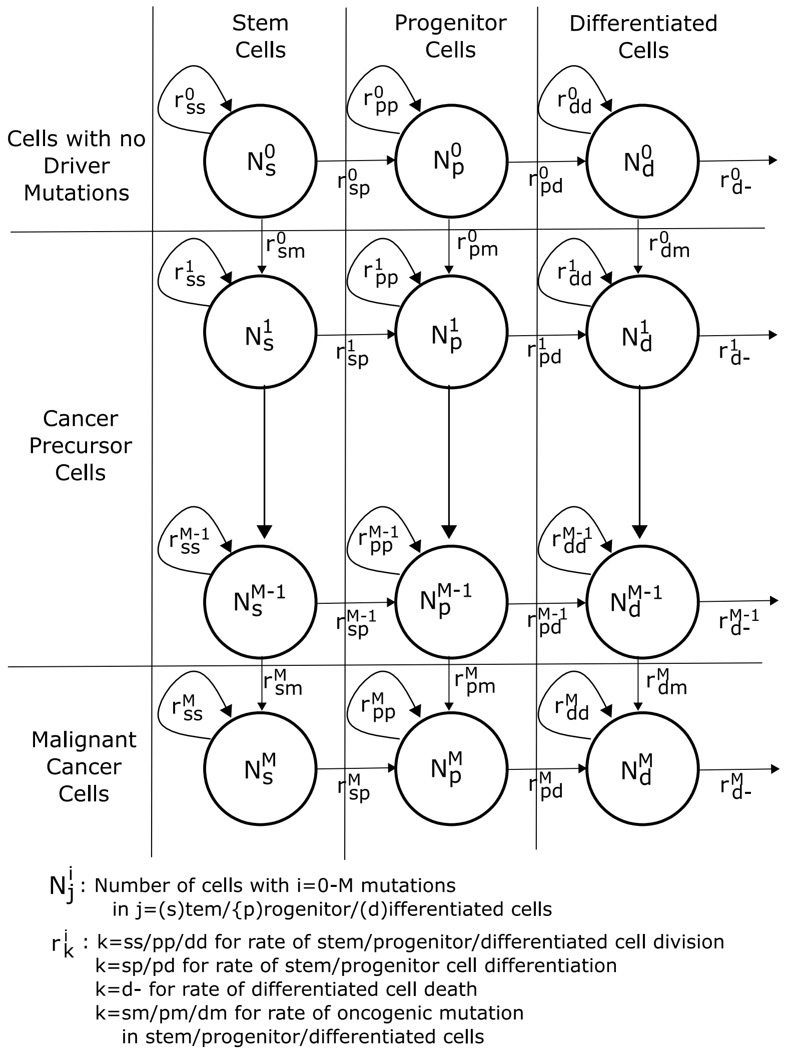

Figure S5. *Mechanistic model of tumor growth.*

Table S3. *Parameters for mechanistic model of tumor growth.*

Parameter	Colon Adeno-carcinoma	Lung Adeno-carcinoma	Stomach Adeno-carcinoma	Thyroid Carcinoma
N_s^0	2.00E+08	1.22E+09	1.00E+08	6.50E+07
N_d^0	3.00E+10	4.34E+11	1.70E+10	2.00E+10
N_p^0	3.00E+10	4.34E+11	1.70E+10	2.00E+10
r_{sp}	73	0.07	36	0.087
r_{pd}	73	0.07	36	0.087
r_{d-}	45.625	45.625	121.67	0.087
r_{ss}	73	0.07	36	0.087
r_{pp}	72.50	0.0698	35.80	0.0867
r_{dd}	27.40	45.555	85.70	0.000336
r_{mut}	8.30E-06	8.30E-06	8.30E-06	8.30E-06
Estimated # hits	3	3	3	3
Alternate value for r_{mut}	8.30E-06	8.30E-06	5.00E-04	5.00E-04
Estimated # hits	3	3	5	5

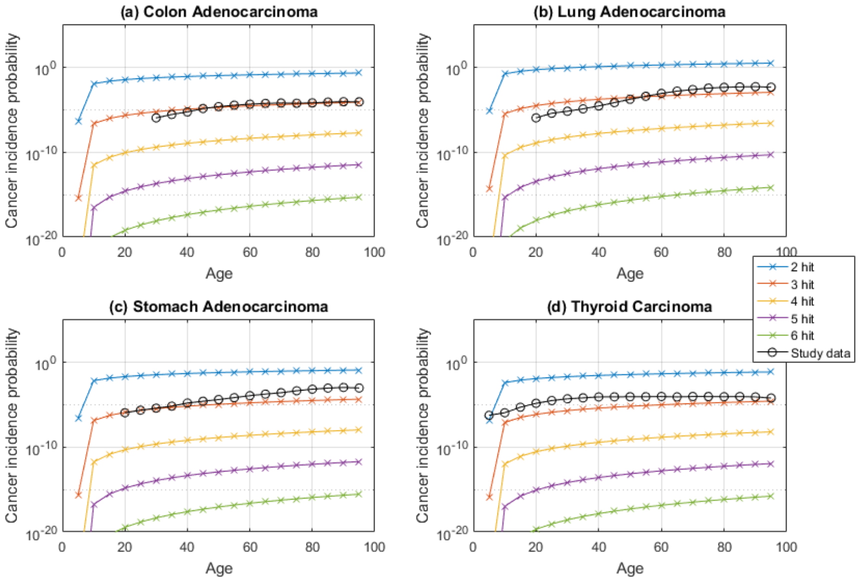

Figure S6. *Cancer incidence probability estimated by mechanistic model and from a recent UK population study data [1]. (a)-(d) Results for four cancer types for which key model parameters were found in the literature. See Table S3.*

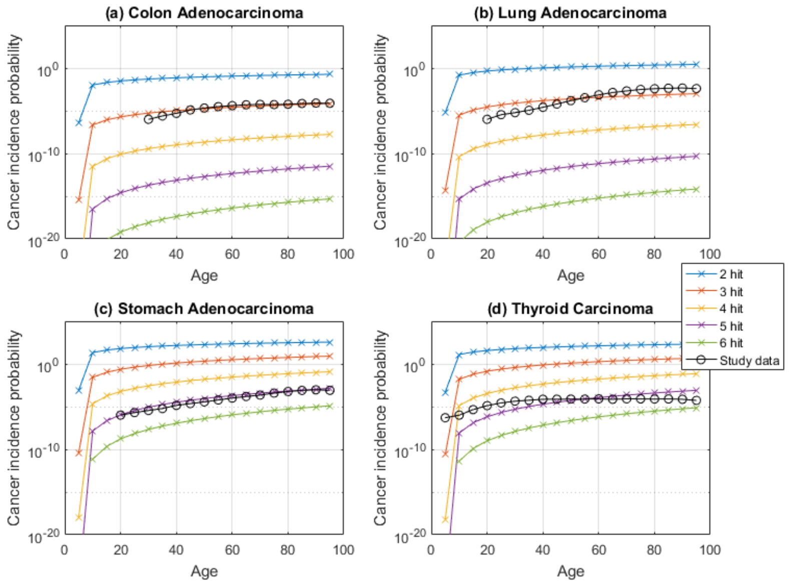

***Figure S7**. Cancer incidence probability estimated by mechanistic model with alternate values for oncogenic mutation rate. (a)-(d) Results for four cancer types for which key model parameters were found in the literature. See Table S3.*

References

1. Cancer Research UK [Internet]. 2019. Available from: https://www.cancerresearchuk.org/health-professional/cancer-statistics/incidence.
2. Werner B, Dingli D, Traulsen A. A deterministic model for the occurrence and dynamics of multiple mutations in hierarchically organized tissues. J R Soc Interface. 2013;10(85):20130349. doi: 10.1098/rsif.2013.0349. PubMed PMID: 23740488; PubMed Central PMCID: PMCPMC4043170.
3. Rodriguez-Brenes IA, Komarova NL, Wodarz D. Cancer-associated mutations in healthy individuals: assessing the risk of carcinogenesis. Cancer Res. 2014;74(6):1661-9. doi: 10.1158/0008-5472.CAN-13-1452. PubMed PMID: 24453004.
4. Weekes SL, Barker B, Bober S, Cisneros K, Cline J, Thompson A, et al. A multicompartment mathematical model of cancer stem cell-driven tumor growth dynamics. Bull Math Biol. 2014;76(7):1762-82. doi: 10.1007/s11538-014-9976-0. PubMed PMID: 24840956; PubMed Central PMCID: PMCPMC4140966.
5. Fillon M. The mathematics of cancer metastases. J Natl Cancer Inst. 2013;105(2):75-6. doi: 10.1093/jnci/djs642. PubMed PMID: 23303865.
6. Altrock P, Liu L, Michor F. The mathematics of cancer: integrating quantitative models. Nature Reviews Cancer. 2015;15(12):730-45. doi: doi: 10.1038/nrc4029.
7. Tomasetti C, Li L, Vogelstein B. Stem cell divisions, somatic mutations, cancer etiology, and cancer prevention. Science. 2017;355(6331):1330-4. doi: 10.1126/science.aaf9011. PubMed PMID: 28336671.
8. Flindt R. Amazing numbers in biology. Berlin: Springer-Verlag; 2006. xiv, 295 p. p.
9. Bertalanffy FD, Nagy KP. Mitotic activity and renewal rate of the epithelial cells of human duodenum. Acta Anat (Basel). 1961;45:362-70. PubMed PMID: 13868378.
10. Coclet J, Foureau F, Ketelbant P, Galand P, Dumont JE. Cell population kinetics in dog and human adult thyroid. Clin Endocrinol (Oxf). 1989;31(6):655-65. PubMed PMID: 2627756.
11. Nunney L, Muir B. Peto's paradox and the hallmarks of cancer: constructing an evolutionary framework for understanding the incidence of cancer. Philos Trans R Soc Lond B Biol Sci.

2015;370(1673). doi: 10.1098/rstb.2015.0161. PubMed PMID: 26056359; PubMed Central PMCID: PMCPMC4581038.
12. Hornsby C, Page K, Tomlinson I. What can we learn from the population incidence of cancer? Armitage and Doll revisited. The Lancet Oncology. 2007;8(11):1030-8. doi: 10.1016/s1470-2045(07)70343-1.
13. Knudson A. Mutation and Cancer: Statistical Study of Retinoblastoma. Proc Natl Acad Sci U S A. 1971;68(4):820-3.
14. Ashley DJ. The two "hit" and multiple "hit" theories of carcinogenesis. Br J Cancer. 1969;23(2):313-28.
