# Supplementary figures and images for: Estimating the number of genetic mutations (hits) required for carcinogenesis based on the distribution of somatic mutations
Source: PLoS Comput Biol. 2019 Mar 7;15(3):e1006881. doi: 10.1371/journal.pcbi.1006881 (PMC6424461; doi:10.1371/journal.pcbi.1006881)

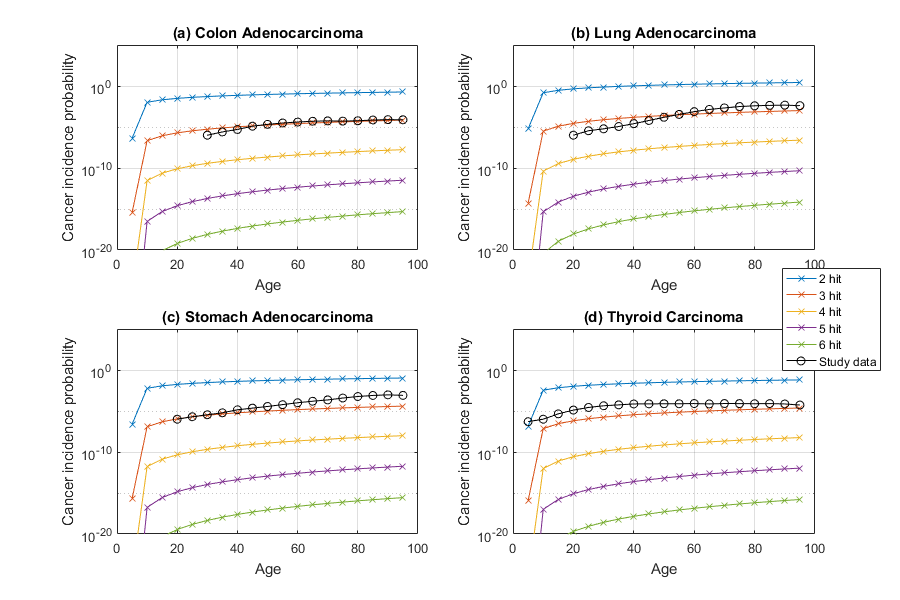

Supplement: S6 Fig — (a)- (d) Results for four cancer types for which key model parameters were found in the literature. (TIF) [file pcbi.1006881.s007.tif]

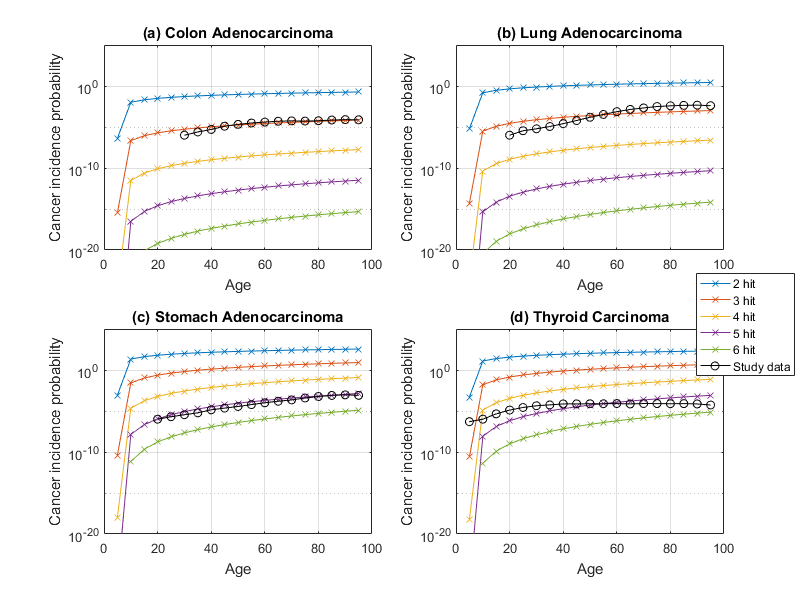

Supplement: S7 Fig — (a)- (d) Results for four cancer types for which key model parameters were found in the literature. (TIF) [file pcbi.1006881.s008.tif]
