# Supplementary material for: Estimating the number of genetic mutations (hits) required for carcinogenesis based on the distribution of somatic mutations
Source: PLoS Comput Biol. 2019 Mar 7;15(3):e1006881. doi: 10.1371/journal.pcbi.1006881 (PMC6424461; doi:10.1371/journal.pcbi.1006881)
Supplement: S1 Table — For sample size greater than 200, there is no difference in number of hits between results for all samples and randomly selected 80% of samples, and the number of combinations is different in only one case. Although there are no differences in the number of hits for 100–200 samples, the RMSD in many cases is large, due to significant discontinuity in the distribution. (DOCX) [file pcbi.1006881.s009.docx]

**Table S1. Results are robust for sample size greater than 200**. For sample size greater than 200, there is no difference in number of hits between results for all samples and randomly selected 80% of samples, and the number of combinations is different in only three cases. Although there are no differences in the number of hits for 100-200 samples, the RMSD in many cases is large, due to significant discontinuity in the distribution.
