# Supplementary material for: Estimating the number of genetic mutations (hits) required for carcinogenesis based on the distribution of somatic mutations
Source: PLoS Comput Biol. 2019 Mar 7;15(3):e1006881. doi: 10.1371/journal.pcbi.1006881 (PMC6424461; doi:10.1371/journal.pcbi.1006881)
Supplement: S2 Table — The estimated number of hits are the same when G is 8 times the value used for the results shown in Tables 1 and S1, except for uterine carcinosarcoma (UCS). (DOCX) [file pcbi.1006881.s010.docx]

**Table S2. Results are robust for different values of G, the number of possible mutations**. The estimated number of hits are the same when G is 8 times the value used for the results shown in Tables 1 and S1, except for uterine carcinosarcoma (UCS).
