# Supplementary material for: Estimating the number of genetic mutations (hits) required for carcinogenesis based on the distribution of somatic mutations
Source: PLoS Comput Biol. 2019 Mar 7;15(3):e1006881. doi: 10.1371/journal.pcbi.1006881 (PMC6424461; doi:10.1371/journal.pcbi.1006881)
Supplement: S3 Table — (DOCX) [file pcbi.1006881.s011.docx]

**Table S3**. Parameters for mechanistic model of tumor growth.

| Parameter | Colon Adeno-carcinoma | Lung Adeno-carcinoma | Stomach Adeno-carcinoma | Thyroid Carcinoma |
| --- | --- | --- | --- | --- |
| *N_s_^0^* | 2.00E+08 | 1.22E+09 | 1.00E+08 | 6.50E+07 |
| *N_d_^0^* | 3.00E+10 | 4.34E+11 | 1.70E+10 | 2.00E+10 |
| *N_p_^0^* | 3.00E+10 | 4.34E+11 | 1.70E+10 | 2.00E+10 |
| *r_sp_* | 73 | 0.07 | 36 | 0.087 |
| *r_pd_* | 73 | 0.07 | 36 | 0.087 |
| *r_d-_* | 45.625 | 45.625 | 121.67 | 0.087 |
| *r_ss_* | 73 | 0.07 | 36 | 0.087 |
| *r_pp_* | 72.50 | 0.0698 | 35.80 | 0.0867 |
| *r_dd_* | 27.40 | 45.555 | 85.70 | 0.000336 |
| *r_mut_* | 8.30E-06 | 8.30E-06 | 8.30E-06 | 8.30E-06 |
| Estimated # hits | 3 | 3 | 3 | 3 |
|  |  |  |  |  |
| Alternate value for *r_mut_* | 8.30E-06 | 8.30E-06 | 5.00E-04 | 5.00E-04 |
| Estimated # hits | 3 | 3 | 5 | 5 |
